# The Effects of Biostimulants, Biofertilizers and Water-Stress on Nutritional Value and Chemical Composition of Two Spinach Genotypes (*Spinacia oleracea* L.)

**DOI:** 10.3390/molecules24244494

**Published:** 2019-12-08

**Authors:** Carla Pereira, Maria Inês Dias, Spyridon A. Petropoulos, Sofia Plexida, Antonios Chrysargyris, Nikos Tzortzakis, Ricardo C. Calhelha, Marija Ivanov, Dejan Stojković, Marina Soković, Lillian Barros, Isabel C.F.R. Ferreira

**Affiliations:** 1 Centro de Investigação de Montanha (CIMO), Instituto Politécnico de Bragança, Campus de Santa Apolónia, 5300-253 Bragança, Portugal; carlap@ipb.pt (C.P.); maria.ines@ipb.pt (M.I.D.); calhelha@ipb.pt (R.C.C.); lillian@ipb.pt (L.B.); 2 Crop Production and Rural Environment, Department of Agriculture, University of Thessaly, 38446 N. Ionia, Magnissia, Greece; sofiaplexida@uth.gr; 3 Biotechnology and Food Science, Department of Agricultural Sciences, Cyprus University of Technology, 3036 Lemesos, Cyprus; a.chrysargyris@cut.ac.cy (A.C.); nikolaos.tzortzakis@cut.ac.cy (N.T.); 4 Department of Plant Physiology, Institute for Biological Research “Siniša Stanković”-National Institute of Republic of Serbia, University of Belgrade, Bulevar Despota Stefana 142, 11000 Belgrade, Serbia; mris@ibiss.bg.ac.rs (M.I.); dejanbio@ibiss.bg.ac.rs (D.S.); marija.smiljkovic@ibiss.bg.ac.rs (M.S.)

**Keywords:** antimicrobial properties, antioxidant activities, bioactive properties, biostimulants, oxalic acid, phenolic compounds, spinach, *Spinacia oleracea* L., tocopherols, water stress

## Abstract

In the present study, the effect of biostimulants application on the nutritional quality and bioactive properties of spinach cultivated in protected environment under water stress conditions was evaluated. For this purpose, four commercially available biostimulant products (Megafol (MEG), Aminovert (AM), Veramin Ca (V), Twin Antistress (TA), and two spinach genotypes (Fuji F1 and Viroflay) were tested under two irrigation regimes (normal irrigation (W+), and water-holding (W–). Fat and carbohydrates content was favored by water stress when Megafol (MEGW+) and Veramin (VW+) were applied on Fuji plants, while calorific value was also increased by MEGW+ treatment. In contrast, protein and ash content increased when AMW– and TAW+ were applied on Viroflay plants. Raffinose and glucose were the most abundant sugars, followed by sucrose and fructose, with the highest contents recorded for Fuji plants when AMW+ (fructose, glucose and total carbohydrates), CW– (sucrose), and TAW– (raffinose) treatments were applied. Regarding organic acids, oxalic and malic acid which had the highest contents for the TAW– (Viroflay plants) and AMW– (Fuji plants) treatments, respectively. α- and *γ*-tocopherol were the only isoforms detected with MEGW– and VW– inducing the biosynthesis of *α*-tocopherol, while AMW+ increased *γ*-tocopherol content in Fuji plants. The main fatty acids were *α*-linolenic and linoleic acids which were detected in the highest amounts in AMW–, AMW+, and TAW+ the former and in AMW–, VW–, and CW+ the latter. Regarding phenolic compounds content, peak 12 (5,3′,4′-Trihydroxy-3-methoxy-6:7-methylenedioxyflavone-4′-glucuronide) was the most abundant compound, especially in Viroflay plants under normal irrigation and no biostimulants added (CW–). The antioxidant and cytotoxic activity of the tested samples did not show promising results when compared with the positive controls, while a variable antibacterial activity was recorded depending on the tested biostimulant, irrigation regime and genotype. In conclusion, a variable effect of the tested biostimulants and irrigation regimes was observed on bioactive properties and chemical composition of both spinach genotypes which highlights the need for further research in order to make profound conclusions regarding the positive effects of biostimulants under water stress conditions.

## 1. Introduction

Global warming and the prolonged dry periods along with the untimely weather conditions due to the climate change (CC) have raised the scientific research and multiple studies have been carried out to understand, explain and prevent their detrimental side effects on living organisms and the environment [1,2,3]. In addition, the scarcity of irrigation water in the semi-arid and arid regions of the Mediterranean basin is becoming the major barrier for crop production. Special focus is given on production of vegetables which have high irrigation requirements compared to other crops and yield components which are greatly affected by water stress conditions [4]. Several strategies have been suggested to overcome this major issue, including deficit irrigation or water-holding irrigation regimes. Other strategies include the use of alternative water sources (treated-waste water, saline water etc.), the selection of less water demanding crops or tolerant genotypes, and cultivation practices. Recently the use of biostimulants and biofertilizers has also attracted great interest from the market and the research community [5,6,7]. Especially for the use of biostimulants in agriculture, the related farming sector is facing an enormous growth with several new products having been released and extensive research being carried out focusing on the effects of biostimulants on yield and quality parameters of crops [8,9,10].

Plant biostimulants could be considered as those compounds that may promote plant growth, increase tolerance to abiotic stressors and at the same time improve natural resources use efficiency [6,11,12]. In this context, various natural compounds could be included in this group such as beneficial microorganisms, protein hydrolysates, humic acids, seaweed extracts, and amino acids [13,14,15,16]. These compounds are usually classified according to their source of origin and not based on their chemical structure [11,12]. Biostimulants extracted from food processing by-products is a promising approach for waste management and the circular economy which has been examined in vegetable production with promising results in terms of the yield and quality of the final product [8,17,18].

Biostimulants have the capacity to modify plant physiological processes. Moreover, they can partly substitute the fertilizer use, improve yields, increase resistance to drought, salinity, flooding, and thermal stresses and ultimately have a positive effect on plant growth and physiology [5,19]. They may also play a significant role on the increase of plant resistance to biotic stress from pests and pathogens [20]. While they can alleviate oxidative stress symptoms caused by mineral deficiencies in vegetable crops (e.g., tomato), since they contain amino acids, humic acids, vitamins, and minerals [21,22]. However, a biostimulant’s efficacy is dependent on the application frequency and doses, the plant growth stage and wellness during application, and also on the chemical composition of the diverse commercial products available at the market [17,23,24]. Especially for drought stress, the positive effect of biostimulants on plants is related to the cytokinin content of some algae extracts and humic substances that may increase endogenous cytokinins levels [25,26].

Several reports have suggested that the application of biostimulants on horticultural crops can induce the primary and secondary metabolism of plants thus increasing nutrients use efficiency and modulating nutritional value and chemical composition of the final products [5,9,13]. Considering the increased market demands for higher availability of food products of enhanced nutritional quality, the use of biostimulants has been suggested as an eco-friendly practice for crop production under non-optimal conditions (abiotic stress conditions) to retain high yields without compromising the quality of the final product [13,23,27,28]. Special attention was given to research on leafy vegetables since they are more prone than other horticultural crops to abiotic stressors and drought, thermal, nutrient, and salinity stress in particular [4,19,24,27,29,30].

Spinach (*Spinacia oleracea* L.) is a leafy vegetable crop which is widely cultivated throughout the Mediterranean basin in open-field and protected conditions. It is a common ingredient of many dishes and food products due to the exceptional nutritional value and the important bioactive properties of its leaves [31,32,33]. Apart from beneficial compounds content, spinach may be also rich in antinutrients such as nitrates and oxalates with severe implications on human health that have to be considered, and proper cultivation practices are usually applied to limit the content of such compounds [34,35,36,37]. The beneficial effects of biostimulants application on the yield and quality of spinach through the increase of bioactive compounds’ content and nutritional value were reported in several recent studies [19,27,38,39,40,41]. However, a variable response of spinach to biostimulants application has been suggested depending on growing conditions and biostimulant product which necessitates further studying [13].

Consequently, the aim of the present was to study the effect of four biostimulants application on the chemical composition and bioactive properties of spinach plants grown under water stress conditions. For this purpose, two commercially available spinach genotypes were selected and grown in soil under protected conditions and controlled irrigation regimes to evaluate the potential of using biostimulants as an environmental friendly cultivation strategy to alleviate the negative effects of water stress conditions and to improve the quality of vegetable crops. Moreover, considering the varied composition of the applied biostimulants possible differences in plant response attributed to such variation will be also discussed. 

## 2. Results and Discussion

### 2.1. Nutritional Value and Chemical Composition

#### 2.1.1. Nutritional Value

The nutritional value of the spinach samples was assessed, and the results are presented in Table 1.

In general, Fuji genotype presented higher fat, carbohydrates, and energy content (7.8 ± 1.6 and 37.8 ± 2.6 g/100 g dw, and 364.9 ± 10.3 kcal/100 g dw, respectively) than Viroflay genotype. Interestingly, comparing all the samples from both genotypes, the water stress did not significantly affect the fat and protein content, and energy values, with water-holding irrigation regime leading to increased carbohydrate levels (37.2 ± 2.8 g/100 g dw) and decreased ash contents (18.55 ± 1.02 g/100 g dw). In what concerns biostimulants application regardless of the water regime and the genotype tested, Twin-Antistress was the one presenting the most noticeable effects in the assessed spinach samples, which revealed lower fat and energy values, but higher ash concentration (5.3 ± 1.6 g/100 g dw, 340.3 ± 15.9 kcal/100 g dw, and 21.5 ± 2.6 g/100 g dw, respectively) than the samples treated with the rest of the biostimulants and the control treatment. On the other hand, Veramin Ca supplementation seamed to decrease the protein content in spinach leaves (35.2 ± 1.8 g/100 g dw). The highest fat concentration was found in Fuji genotype treated with Megafol (10.7 ± 0.3 g/100 g dw), while the same combination also presented the highest energetic contribution (383.3 ± 0.9 kcal/100 g dw). On the contrary, the lowest levels were detected in Fuji genotype treated with Twin-Antistress, and the Viroflay genotype supplemented with Aminovert and Twin-Antistress (4.4 ± 0.1, 4.3 ± 0.2, and 4.2 ± 0.2 g/100 g dw, respectively), under water-holding irrigation regime, except for Twin-Antistress treated Viroflay plants. The latter combination was also the one presented the lowest energy value (319.4 ± 0.9 kcal/100 g dw) and the highest ash content (25.4 ± 0.1 g/100 g dw). Fuji genotype treated with Veramin Ca under water-holding irrigation regime revealed increased carbohydrate and decreased protein and ash concentrations (42.9 ± 0.3, 32 ± 1, and 17.4 ± 0.3 g/100 g dw, respectively) than the other samples, with an ash content close to the ones found in the same genotype treated with Megafol under the same water stress conditions and in the non-biostimulant supplemented spinach plants growing under normal irrigation regime (17.53 ± 0.02 and 17.3 ± 0.4 g/100 g dw, respectively). Finally, the highest protein concentration was found in Viroflay plants treated with Aminovert, under water-holding irrigation conditions (42.0 ± 0.3 g/100 g dw). To the best of our knowledge, there are no reports on the effects of the studied biostimulants on spinach cultivation, notwithstanding, a previous study assessing the supplementation of spinach plants with the biostimulant Kelpak (produced from the brown seaweed *Ecklonia maxima* (Osbeck) Papenfuss) where an increased protein level by 16.3% was observed [42]. Moreover, the treatment with Trainer (legume-derived protein hydrolysate), Kelpak, and Amalgerol (plant extracts, essential oils and fatty acids in oil/water emulsion, while extracts of the seaweed *Ascophyllum nodosum*) highly influenced the protein concentration of spinach samples, which was higher by 12.4% than in untreated plants [13]. These effects are more pronounced than those observed in the present study, where the maximum recorded increase of protein content was by 7.1%, in Viroflay genotype supplemented with Aminovert under water-holding irrigation regime. This difference could be attributed to the fact that no stress treatments were applied in these studies, as well as to differences in nitrogen fertilization and the nitrogen use efficiency of the tested genotypes which resulted in differences in the protein content [43]. Moreover, in contrast to the study of Rouphael et al. [13] Twin-Antistress which contains *A. nodosum* extracts as the Almagerol product did not have a positive effect on protein content probably due to differences in the overall composition between the two products. On the other hand, Aminovert which contains a high percentage (>80%) of amino acids had a positive effect on protein content in water-stressed Viroflay plants which could be due to an increased activity of nitrate reductase and the nitrogen uptake from roots [12].

#### 2.1.2. Free Sugars

Free sugars composition in spinach leaves is presented in Table 2, where it can be observed that, in general, Fuji spinach leaves revealed higher concentrations of fructose, sucrose, raffinose, and total sugars (1.42 ± 0.03, 2.056 ± 0.03, 2.736 ± 0.08, and 8.66 ± 0.09 g/100 g dw, respectively) than those of Viroflay genotype, whereas glucose was detected in similar amounts in both genotypes (2.46 ± 0.05 and 2.11 ± 0.03 g/100 g dw, respectively). The highest levels of total sugars were found in Fuji genotype supplemented with Aminovert under normal irrigation regime (10.74 ± 0.02 g/100 g dw), which also presented improved levels of fructose and glucose. Interestingly, this treatment did not affect the fructose content in Viroflay genotype, under the same irrigation conditions (normal), which presented the lowest levels of this free sugar, along with the control sample (0.222 ± 0.003 and 0.244 ± 0.002 g/100 g dw, respectively). On the other hand, the lowest total sugar concentrations were detected in Viroflay plants treated with Megafol (4.96 ± 0.05 g/100 g dw) and Veramin Ca (4.87 ± 0.09 g/100 g dw), under water-holding and normal irrigation regime, respectively. Twin-Antistress supplementation resulted in the highest (3.34 ± 0.08 g/100 g dw) and the lowest (1.48 ± 0.04 g/100 g dw) raffinose levels, which were quantified in Fuji plants with water-holding irrigation regime and in Viroflay with normal irrigation conditions, respectively. Considering all the tested biostimulants, Twin-Antistress and Aminovert revealed to enhance glucose production (2.66 ± 0.03 and 2.58 ± 0.03 g/100 g dw, respectively). Aminovert treatment did not affect raffinose levels (2.67 ± 0.02 g/100 g dw) when compared to the control samples (2.79 ± 0.08 g/100 g dw), while the other biostimulants induced lower concentrations of this sugar, especially the Twin-Antistress treatment. The application of biostimulants did not significantly affect fructose, sucrose, nor total sugars content. Fructose and total sugars were not significantly affected by water stress conditions, whereas sucrose and raffinose production was favored by water-holding irrigation regime (1.71 ± 0.06 and 2.743 ± 0.001 g/100 g dw, respectively), and glucose was detected in higher quantity in spinaches subjected to normal irrigation conditions (2.51 ± 0.03 g/100 g dw). When testing the combinatory effect of the tested factors, the supplementation of Aminovert on Fuij water-stressed plants resulted in the highest content of fructose, glucose and total sugars content, while sucrose was the highest in normally irrigated plants with no biostimulants added and raffinose in normally irrigated plants treated with Twin-Antistress or no biostimulants added. As far as we know, there are no previous reports on the effect of biostimulants application on free sugars in spinach plants since most of the studies refer to total soluble sugars content. However, according to Rathinasabapathi water stress induces the biosynthesis of free sugars which may act as osmoprotectants and this trend was also observed in our study without significant differences between normally irrigated and water-stressed plants [44]. The increasing trends observed in water-stressed plants could be also attributed to the fact that soluble sugars are involved in the regulation of stress and growth related genes and in plant defense mechanisms while they are also utilized by the plants as carbon and energy pools [40,45]. In the study of Goñi et al. [46], the application of three different *Ascophyllum nodosum* extracts had a variable effect on soluble sugars content in tomato leaves depending on extract composition and harvesting time, while drought stress also induced sugars accumulation in plant leaves comparing to normally irrigated plants. Similarly, Twin-Antistress increased the accumulation of raffinose under water-holding conditions but only in the case of Fuji plants. Moreover, Gutiérrez-Rodríguez et al. [47] reported significant differences in non-cellulosic neutral sugars between two spinach cultivars (cv. Whale and Bolero), a trend which was also observed in our study where Fuji plants had a high total and individual sugars content regardless of the irrigation regime and the biostimulant application.

#### 2.1.3. Organic Acids

The results obtained for organic acids analysis in spinach samples are presented in Table 3. All the assessed samples revealed oxalic and malic acids as the most abundant organic acids, and residual concentrations of fumaric acid. Ascorbic acid was also detected in low concentrations in some samples, being more abundant in spinach plants cultivated under normal irrigation conditions (0.0108 ± 0.0001 g/100 g dw) and in Fuji genotype (0.0097 ± 0.0001 g/100 g dw). Malic acid was also predominant in this genotype (2.951 ± 0.007 g/100 g dw), and in plants cultivated under water-holding conditions (2.803 ± 0.005 g/100 g dw). The application of biostimulants did not affect malic acid concentration but revealed to decrease the levels of ascorbic and total organic acids and, in some cases, those of oxalic acid (Aminovert and Twin-Antistress treated samples). In terms of the combinatory effect of the tested factors, both malic and ascorbic acids were found in higher amounts in Fuji genotype supplemented with Aminovert under water-holding irrigation regime (3.6 ± 0.1 g/100 g dw) and without biostimulants supplementation (control sample; 0.0183 ± 0.0007 g/100 g dw) under normal irrigation conditions, whereas oxalic acid was abundant in Viroflay spinaches treated with Twin-Antistress under the normal irrigation regime (5.15 ± 0.02 g/100 g dw). On the other hand, the lowest levels of oxalic and malic acids were quantified in spinaches treated with Twin-Antistress, namely in Fuji under water-holding irrigation and Viroflay subjected to normal irrigation (3.3 ± 0.1 and 1.73 ± 0.02 g/100 g dw, respectively). The lack of previous studies on the effect of biostimulants application on organic acids composition in spinach leaves does not allow us to compare the results obtained herein with the ones presented by other authors. However, studies in other leafy vegetables reported a significant effect of biostimulants on oxalic acid content and no effects on malic acid content in perennial wall rocket (*Diplotaxis tenuifolia* (L.) DC.) leaves [48], which was also recorded in our study. Moreover, considering that ascorbic acid is a precursor of oxalic acid in oxalate-accumulating plants could partly justify the fact that oxalic acid content was similar to the control treatment in Megafol-treated plants where the lowest ascorbic acid content was observed [49]. The composition of biostimulants may also have a significant effect on organic acids composition, since organic acids and oxalic acid in particular are highly involved in microorganisms metabolism (*quod vide* Twin-Antistress which contains *Bacillus subtilis* and yeast) [50]. Another possible explanation for the effect of biostimulant composition on oxalic acid content would be related with Ca application (*quod vide* Veramin Ca) which is associated with calcium oxalate formation for the removal of excessive calcium or oxalic acid [50].

#### 2.1.4. Tocopherols

Regarding tocopherols composition (Table 4), all the spinach samples contained α and γ isoforms. In general, Fuji spinaches revealed higher concentrations of these compounds, with samples growing under water-holding irrigation regime being richer in α and total tocopherols, while Viroflay genotype revealed a higher concentration of γ-tocopherol (0.989 ± 0.003 mg/100 g dw). This isoform was not significantly affected by water stress conditions, contrarily to α and total tocopherols, which were predominant in spinaches growing under water-holding regime (8.117 ± 0.005 and 8.975 ± 0.003 mg/100 g dw, respectively). The biostimulants application increased α- and total tocopherols, especially in the case of Megafol (8.46 ± 0.08 and 9.35 ± 0.02 mg/100 g dw, respectively), Veramin Ca (8.59 ± 0.03 and 9.49 ± 0.04 mg/100 g dw, respectively) and Aminovert (7.44 ± 0.01 and 8.48 ± 0.02 mg/100 g dw, respectively), whereas Twin-Antistress decreased both isoforms concentration, when compared to the non-treated samples. The same trend was observed in water-stressed Fuji plants when Megafol and Aminovert were applied. The lowest levels of tocopherols were detected in Twin-Antistress supplemented Viroflay samples, both in water-holding (γ-tocopherol; 0.58 ± 0.02 mg/100 g dw) and normal irrigation regime (α and total tocopherols; 3.21 ± 0.02 and 3.92 ± 0.02 mg/100 g dw, respectively). Although α-tocopherol has been already reported in spinach leaves [51,52], to the best of our knowledge, the effect of biostimulants application on tocopherols content in spinach plants and leafy vegetables in general has not been previously assessed. Moreover, the overall increase of *α*- and total tocopherols under water-holding conditions is associated with the plant defense mechanisms against abiotic stressors [53,54] and stress recovery processes [55]. On the other hand, Ca supplementation may also up-regulate the content of non-enzymatic antioxidants such as tocopherols [56,57], while nitrogen containing-compounds such as amino acids and tocopherols were associated with the increased efficiency of sunflowers against drought stress [58].

#### 2.1.5. Fatty Acids

Twenty-six fatty acids were detected in the spinach samples (Table 5 and Table 6), although not all of them were present in all the analyzed samples. α-linolenic and linoleic acids were the most abundant fatty acids regardless of the tested treatment accounting for more than 81% of total detected fatty acids, while polyunsaturated fatty acids was the most abundant class (83.3%–87.9%) due to the rich content of α-linolenic acid (66.44%–75.7%). Similar results regarding the composition and classification of fatty acids in spinach leaves were reported by Zemanová et al. [59] and Maeda et al. [60] who highlighted the rich content of spinach leaves in polyunsaturated and omega-3 fatty acids in particular. In what concerns the effects of single factors on the fatty acid classes and the main fatty acids content (the statistical analysis is not presented), saturated and monounsaturated fatty acids content was higher in Viroflay than Fuji plants, whereas the opposite trend was recorded for polyunsaturated fatty acids. Moreover, no differences were observed in the fatty acid classes regarding the effect of water stress, as well as for the saturated fatty acids when considering the application of the various biostimulants. However, both monounsaturated and polyunsaturated fatty acids were affected by the biostimulant application, with Megafol and Aminovert-treated plants showing the highest content of monounsaturated and polyunsaturated fatty acids, respectively. Fuji plants contained higher amounts of α-linolenic acid than Viroflay, whereas the opposite trend was observed for linoleic acid. Neither water regime nor biostimulant application affected α-linolenic acid, while linoleic acid content was higher when plants were treated with Megafol, Twin-Antistress, and Aminovert. Regarding the combinatory effects of the tested factors (Table 5 and Table 6), Viroflay samples treated with Megafol under normal irrigation conditions revealed the highest levels of saturated (13.60 ± 0.04%) and monounsaturated (4.9 ± 0.1%) fatty acids, while the highest concentrations of polyunsaturated fatty acids were found in Fuji genotype control samples cultivated under normal irrigation regime (87.85 ± 0.02%) and Aminovert supplemented samples subjected to water-holding irrigation regime (87.9 ± 0.2%). Megafol treatment increased the concentrations of caprylic (0.033 ± 0.001%), capric (0.111 ± 0.001%), lauric (0.132 ± 0.008%), myristic (0.59 ± 0.01%), pentadecylic (0.154 ± 0.005%), palmitic (9.57 ± 0.04%), margaric (0.082 ± 0.001%), stearic (1.282 ± 0.001%), and oleic (3.086 ± 0.006%) acids in Viroflay genotype growing under normal irrigation conditions. The production of behenic, docosahexaenoic, tricosylic, and lignoceric acids (0.51 ± 0.02, 0.169 ± 0.002, 0.103 ± 0.002, and 1.34 ± 0.02%, respectively) in the same genotype was favoured by the application of Twin-Antistress biostimulant. Aminovert treated Fuji plants (water-holding irrigation regime) revealed the highest concentrations of docosahexaenoic and α-linolenic acids (0.218 ± 0.006 and 75.7 ± 0.2%, respectively), with this latest being present in similar quantities in the control treatment (normal irrigation; 75.2 ± 0.2%) and Veramin Ca supplemented plants (water-holding irrigation; 75.1 ± 0.3%). Viroflay spinaches presented the highest levels of linoleic acid, especially in Aminovert treated samples, both under normal and water-holding irrigation (15.5 ± 0.8 and 16 ± 1%, respectively), and Twin-Antistress application with normal irrigation (15.3 ± 0.2%). The lack of previous similar studies limited the comparison of these results with those obtained in other researches. However, unsaturated fatty acids accumulation is associated with membrane fluidity and adaptation mechanism to stress conditions. In particular, high contents of α-linolenic acid confer increased tolerance to abiotic stressors such as salinity and drought stress [61]. Moreover, the application of humic-like substances was shown to increase fatty acids content in microalgae, without however any postulations regarding the mechanisms of action being reported [62]. Amino acids application may also have an effect on fatty acids content, since specific amino acids synthesized in plants such as Leucine, Isoleucine, and Valine contain an a branched aliphatic chain and their degradation produces acetyl-CoA which is a substrate for fatty acids biosynthesis [63].

### 2.2. Phenolic Composition

The chromatographic characteristics and tentative identification of the phenolic compounds found in the hydroethanolic extracts obtained from spinach leaves are present in Table 7. Thirteen compounds were tentatively identified, including twelve flavonoids (glucuronides and acylated di- and triglycosides of methylated and methylene dioxide derivatives of 6-oxygenated flavanols) and one phenolic acid (*p*-coumaric acid derivative). All tentatively identified flavonoids have already been extensively described in spinach by other authors [64,65,66,67], being patuletin ([M − H]^−^ at *m/z* 331), spinacetin ([M − H]^−^ at *m/z* 345), and jaceidin ([M − H]^−^ at *m/z* 359) the most characteristics aglycones, while their presence is even considered a determining factor for spinach quality [65]. Two other flavonoids have also been tentatively identified as 5,3′,4′-trihydroxy-3-methoxy-6:7-methylenedioxyflavone-4′-glucuronide and 5,4′-dihydroxy-3,3′-dimethoxy-6:7-methylenedioxiflavone-4′-glucuronide [64,65,66,67]. The presence of quercetin, isorhamnetin, apigenin, luteolin, among others (the most common flavonols found in plants) has been also reported in spinach (in different plant parts and maturity stages), but their presence is in very low amounts, thus they were not identified in the samples studied herein. Regarding the phenolic acid detected in our study, to the best of our knowledge this is the first time that it has been described in spinach leaves, being tentatively identified as 5-*p*-coumaroylquinic acid ([M − H]− at *m/z* 337) based on HPLC retention time and MS^2^ fragmentation characteristics, as previously reported by Clifford et al. [68,69].

Regarding the quantification of the identified phenolic compounds (Table 8), all the studied samples showed a similar phenolic profile, being the genotype Viroflay the one presenting higher amounts of total phenolic acids (TPA; 13.69 ± 0.07 mg/g dw in the Aminovert treatment under normal irrigation conditions), total flavonoids (TF; 141.2 ± 0.6 mg/g dw in the control treatment under water-holding irrigation conditions), and total phenolic compounds (TPC; 148.0 ± 0.6 mg/g dw in the control treatment under water-holding irrigation conditions). The most predominant peak in all the samples was peak 12 (5,3′,4′-trihydroxy-3-methoxy-6:7-methylenedioxyflavone-4′-glucuronide) which is in accordance with the results reported by Bottino et al. [66], where this compound represented 25% of the total flavonoids in the fresh material (major compound), whereas herein it represents up to 56% of the total flavonoids in the dried material. Peaks 4 (patuletin-3-*O*-β-d-(2-ρ-coumaroylglucopyranosyl-(1→6)-[β-d-apiofuranosyl-(1→2)]-β-d-glucopyranoside isomer), 5 (patuletin-3-*O*-β-d-(2-ρ-coumaroylglucopyranosyl-(1→6)-[β-D-apiofuranosyl-(1→2)]-β-d-glucopyranoside), 7 (Spinacetin-3-*O*-β-d-(2-ρ-coumaroyl glucopyranosyl-(1→6)-[β-d-apiofuranosyl-(1→2)]-β-d-glucopyranoside isomer), and 8 (spinacetin-3-*O*-β-d-(2-ρ-coumaroyl glucopyranosyl-(1→6)-[β-d-apiofuranosyl-(1→2)]-β-d-glucopyranoside) were only found in trace amounts in all the tested samples (except for peak 5 that was detected in higher amounts than the limit of quantitation (LOQ) in Fuji genotype treated with Veramin under with normal irrigation regime).

Regarding the treatments performed to the crops, when individually compared it is verified that a water-holding irrigation regime leads to a higher accumulation of phenolic compounds in the control samples. This type of horticultural practice has been already extensively studied showing promising results as a mean to increase the phenolic content in fruit [70], since these secondary metabolites arise precisely in response to stress factors, such as drought stress. The differences between the irrigation treatments regarding the accumulation of phenolic compounds are so significant, that was possible to observed an increase of more than 2 times of total phenolic compounds in the controls of Viroflay genotype (control with water-holding irrigation system = 148.0 ± 0.6 mg/g dw, control with normal irrigation system = 62.1 ± 0.1 mg/g dw). In Fuji plants, the differences were not so profound, but it was also possible to observe an increase of more than 1 time of total phenolic content (control with water-holding irrigation system = 94.4 ± 0.5 mg/g dw, control with normal irrigation system = 77.1 ± 0.2 mg/g dw).

It was possible to observe that the tested treatments did not have a similar effect on both genotypes indicating a genotype-dependent response, when considering the influence of biostimulant on the concentration of individual phenolic compounds. Regarding Fuji genotype, Megafol and Veramin Ca had a positive influence in the concentration of phenolic compounds especially in peaks 1/2/6 and TF/TPC and 10/11/12/13 and TPA, respectively, when conjugated with water-holding; whereas in the normal irrigation system the biostimulant Twin-Antistress stands out for peaks 2/3/11/13 and TPA, and Veramin Ca for peaks 1/5/6/9/10/12 and TF/TPC. It is known that the amino-acids, glycosides, polysaccharides, and organic acids present in Megafol can act as precursors or activators of some phytohormones in plants that could lead to the increase of phenolic compounds [71]. In Viroflay genotype the Aminovert biostimulant stands out both in water-holding and normal irrigation systems; it was observed, comparing the results with the rest of the biostimulants treatments and not with the control, a higher content for TPA (10.0 ± 0.1 and 13.69 ± 0.07 mg/g dw in water-holding and normal irrigation system, respectively), TF (76.4 ± 0.1 and 118.6 ± 0.1 mg/g dw in water-holding and normal irrigation system, respectively) and TPC (86.4 ± 0.2 and 132.25 ± 0.07 mg/g dw in water-holding and normal irrigation system, respectively). Megafol has also a positive effect on the accumulation of some phenolic compounds, but with a much smaller impact, while the same trend was recorded for Twin-Antistress and Veramin Ca too. As far as the authors know, there are no reports about the effect of the biostimulants studied herein on the production of spinach, however similar studies have been conducted for other vegetable crops, such as green beans (*Phaseolus vulgaris* L.) [10]. In this study, Petropoulos et al. [10] studied the effect of Veramin and Twin-AntiStress, and two other biostimulants, Nomoren (20% of arbuscular mycorrhizal fungi (Glomus spp.)) and EKOprop (arbuscular mycorrhizal fungi, rhizosphere symbiotic bacteria (*Bacillus* spp., *Streptomyces* spp., *Pseudomonas* spp., 1.6 × 10^9^ CFU/g in total), and saprophytic fungi, (Trichoderma spp., 5 × 10^5^ CFU/g)) under normal irrigation and water stress conditions, concluding that the application of these biostimulants had a positive effect on the increase of total phenolic compounds content compared with the control treatment (no biostimulants added), especially in seeds of water-stressed plants, whereas significant differences were also observed between the biostimulant treatments, coinciding with the results obtained herein. In another study, Megafol has also been used in pepper fruit (*Capsicum annuum* L., Solanaceae) production in combination with other biostimulants, namely Radifarm (Amino acids, glycosides, polysaccharides, and organic acids),Viva (33% of organic matter, folic acid, vitamins B1, B2, B6, and PP, inositol and humic acids), and Benefit (Organic carbon, amino acids, nucleotides, free enzymatic proteins and vitamins), reaching to the conclusion that the total phenolic content had been significantly increased after the biostimulant conjugation treatment [71]. Moreover, other biostimulants have already been reported to increase the concentration of phenolic compounds (free sinapic acid), such as karrikinolide (KAR1 from smoke) and eckol (from the seaweed *Ecklonia maxima*) in *Spinacia oleracea* L. var Viroflay from South Africa [42].

When analyzing the combined effect of the biostimulant with the irrigation system, the observed results are more dispersed and do not allow a general conclusion about the ideal conjugation to obtain a higher content in phenolic compounds, except in Viroflay genotype with normal irrigation system combined with Aminovert biostimulant where a higher content of TPA, TF, and TPC was obtained, when compared to the water-holding irrigation regime in this genotype. Xu and Leskovar [19] studied also the combined effect of a biostimulant based on *A. nodosum* seaweed extracts and drought stress production system on the quality of spinach (cv. Bloomsdale from USA), reaching to similar conclusions regarding the increasing of phenolic content under water-holding conditions; however the application of the biostimulant did not affect the concentration of phenolic compounds, which is not in accordance with the results obtained herein. Especially for Megafol, it has been reported that its application is associated with drought stress alleviation as indicated by physiological responses and genes expression of tomato plant to water stress conditions [72], although Saa et al. [73] suggested the complexity of studies involving the application of biostimulants despite the overall promising results.

### 2.3. Bioactive Properties

#### 2.3.1. Antioxidant Activity

The results of the cell-based antioxidant activity assays are presented in Table 9. It is possible to observe a great variation between the two methods tested. For the OxHLIA assay only 3 samples revealed antihemolytic activity after 60 min, all revealing lower IC_50_ values than Trolox (positive standard, IC_50_ = 19.6 ± 0.6 µg/mL).

None of the Viroflay genotype samples revealed antihemolytic activity, while for Fuji genotype the normal irrigation system resulted in lower IC_50_ values (VW+ = 187 ± 1 µg/mL) when compared to the water-holding irrigation system (CW– and VW– = 387 ± 11 and 396 ± 10 µg/mL, respectively). For the TBARS assay, all samples revealed the capacity to inhibit the lipid peroxidation, despite the lower IC_50_ value of Trolox (positive standard, IC_50_ = 5.8 ± 0.6 µg/mL). Viroflay genotype did not stand out in any of the treatments, whereas for Fuji genotype the lowest IC_50_ values (after Trolox) were observed in the water-holding irrigation system for plants supplemented with Twin-Antistress (IC_50_ = 94 ± 4 µg/mL) and Veramin Ca (IC_50_ = 85 ± 3 µg/mL) biostimulants. It is important to note that in these samples the application of biostimulants was an important factor for the increase of bioactivity, as these two samples showed an 85–83% bioactivity increase compared to the control (CW–, IC_50_ = 579 ± 2 µg/mL).

Xu and Leskovar [19] reported that the antioxidant capacity of spinach was not significantly altered under a water-holding system; however, the methodologies used by those authors do not allow a direct correlation with the results obtained in the present work. The variance of the obtained results for the antioxidant properties of spinach does not allow to fully correlate them with presence of the phenolic compounds, which is in accordance with the reported by Bottino et al. [66] in fresh-cut spinach. However, Cho et al. [74] reached to the conclusion that flavonoids appear to be the major contributors to antioxidant capacity in spinach. On the other hand, the effectiveness of the extracts is strongly dependent on the antioxidant mechanism tested, which is clearly reflected in the results obtained for the two applied assays (lipid peroxidation and oxidative hemolysis inhibition), similarly to what happens in in vivo systems.

#### 2.3.2. Cytotoxicity and Anti-inflammatory Activity

The results of cytotoxicity and anti-inflammatory activity assays are presented in Table 10, and as it can be observed, only three samples revealed the capacity to inhibit cell growth.

Regarding Fuji genotype samples, only TAW– treatment (water-holding irrigation regime supplemented with Twin-Antistress biostimulant) revealed GI_50_ values for HeLa (GI_50_ = 280 ± 22 µg/mL), HepG2 (GI_50_ = 322 ± 5 µg/mL), and MCF-7 (GI_50_ = 283 ± 11 µg/mL) cell lines. Similarly, in Viroflay plants the MEGW– treatment (water-holding irrigation regime supplemented with Megafol) showed slightly better results than the ones presented by CW+ treatment (control in normal irrigation regime) for HeLa (GI_50_ = 339 ± 5 and 377 ± 17 µg/mL, respectively) and HepG2 (GI_50_ = 364 ± 20 and 383 ± 15 µg/mL, respectively) cell lines. The lack of response of the tested treatments regarding their cytotoxic activities does not allow drawing a definitive conclusion on the possible effect of water-holding or the biostimulant application as cultivation means for the slight increment of bioactivity. Moreover, none of the samples revealed hepatotoxic effects as the GI_50_ values are higher than >400 µg/mL; the same concentrations were observed for the anti-inflammatory activity in all samples. To the best of our knowledge, there is lack of similar reports in the literature regarding the effect of biostimulants on cytotoxic activities of the spinach and leafy vegetables in general which does allow further discussion of the results for the present study. Therefore, further research is needed to consolidate the effects of the combinatory effects of biostimulant and water-holding application as cost-effective practices for the improvement of bioactivities of vegetable products.

#### 2.3.3. Antimicrobial Activity

Regarding the antimicrobial assays, the results for antibacterial and antifungal activities are presented in Table 11 and Table 12, respectively.

For antibacterial activity results (Table 11), the treatments MEGW–, CW+ and VW+ of Fuji genotype revealed promising MIC values against the Gram-positive bacteria *B. cereus* (0.5 mg/mL). Similarly, VW+ (Fuji) also showed promising MIC values against the gram-negative bacteria *E. coli*, *S.* Typhimurium, and *E. cloacae* (0.5 mg/mL). In the case of Viroflay plants, normal irrigation regime samples showed the most promising results, especially in the control treatment (no biostimulants added) against *B. cereus*, *S.* Typhimurium, and *E. cloacae* (0.5 mg/mL). The MEGW+ and TAW+ (Viroflay genotype) also revealed MIC values of 0.5 mg/mL against *B. cereus* and *L. monocytogenes* (only TAW+). For the antifungal activity results (Table 12), none of the samples clearly stood out presenting minimum MIC values of 2 mg/mL, which is considered a very high value in relation to the control and therefore the antifungal activity of the tested samples could not be considered promising. The variability of results also makes it impossible to draw any conclusions as to the individual or combined effect of the irrigation and biostimulant treatment regimen. As far as the author’s knowledge there are no reports on the effect of different irrigation systems and biostimulants on the cytotoxic, hepatotoxic, anti-inflammatory, and antimicrobial properties of spinach. The varied responses obtained from the different irrigation and biostimulant regime samples regarding their bioactive properties showed that these properties could not be associated with the phenolic contents and can be related with other type of bioactive compounds that were not assessed in the present study.

## 3. Materials and Methods

### 3.1. Plant Material and Growing Conditions

The experiment was carried out during the summer-autumn (August-November) of 2017. Seeds of two spinach genotypes, one oriental (Fuji F1: Elanco S.A.; Greece) and one smooth-leafed type (Viroflay: Agrogen S.A.; Greece), were sown in seed trays filled with peat on 10/08/2017. Young seedlings were transplanted at the stage of 3–5 true leaves in the soil of an unheated plastic greenhouse, in Trikala, Greece. The soil at 0–30 cm depth was clay (26% sand, 28% silt, and 46% clay); pH: 8.0 (1:1 soil/H_2_O); organic matter content: 3.1%; CaCO3: 10.8%; available P (Olsen method): 70.9 mg/kg; total N (Kjeldahl method): 1.6 g/kg; exchangeable K_2_O (ammonium acetate method): 195 mg/kg; electrical conductivity (ECe): 0.75 dS/m [10]. 

Transplantation took place on 28/09/2017 at a distance of 25 cm within each row and 30 cm between rows and a plant density of 133300 plants ha^−1^. Each plot was 2 m long and 1.5 m wide (3 m^2^) with 40 plants in each plot (800 plants in total). The experimental layout was set according to the randomized complete block design with three replication per treatment. Three factors were tested, namely genotype, water regime and biostimulants treatments. Biostimulants treatments included: (1) Control (C: no biostimulants added), (2) Twin-Antistress^®®^ (TW; Microspore Hellas—Sacom Hellas, Greece), (3) Veramin Ca^®®^ (V; Microspore Hellas—Sacom Hellas, Greece), (4) Megafol^®®^ (MEG; Valagro S.p.A, Spain), and (5) Aminovert^®®^ (AM: Luca Viano S.A., Italy). 

Twin-Antistress contains natural microorganisms (*Bacillus subtilis* and yeast) and *Ascophyllum nodosum* extracts, as well as N (organic): 1%, organic carbon: 10%, and organic matter (<50 kDa): 30%; Veramin Ca contains a complex of amino acid of vegetable origin and *Aloe vera* extract, and CaO: 15.6%; Megafol contains Ν (organic): 3.6%, Κ_2_Ο 9.7%, Organic matter: 18.9%, betaines, vitamins, amino acids of plant origin, proteins, and growth promoters; Aminovert contains a high percentage (>80%) of free amino acids of vegetal matrix [10,72]. The application of biostimulants was based on the directions for use as indicated on the label of each product, namely (TW): was applied with irrigation water 5 L ha^−1^ for each dose; (V): was applied with foliar spraying at 500 g 100 L^−1^ H_2_O for each dose; (MEG): was applied with foliar spraying at 200 mL 100 L^−1^ H_2_O for each dose; (AM): was applied with foliar spraying at 200 g L^−1^ H_2_O for each dose. The application of biostimulants was performed twice (10 and 20 days after transplantation; DAT)

Water stress treatments have been previously described in the study of Petropoulos et al. [10]. Briefly, they included (i) plants irrigated normally (W+) (the irrigation was applied twice a week in order tensiometer readings (Irrometer-Moisture Indicator, Irrometer, Riverside, CA) be within the range of 10%–15%,) and (ii) plants where water stress (water holding) was applied (W–) (plants were irrigated approximately once a week in order to keep the tensiometer readings above 40%–50%). A drip irrigation system was used with one dripper per plant and a water flow rate of 3.0 L h^−1^ for each dripper (total amount of water: 450 m^3^ ha^–1^ (3.4 L plant^–1^) for normally irrigated plants (W+) and 250 m^3^ ha^−1^ (1.87 L plant^−1^) for water stressed plants (W–). Water holding started biostimulants were applied for the second time (20 DAT).

Harvest took place on 06/11/2017 (40 DAT) and leaves of marketable size and quality were cut above the ground surface. In order to eliminate the borders’ effect all plants adjusting to the corridors were excluded and only the inner plants (18 plants) from each plot were harvested. After cutting, leaves were cleaned with distilled water, let dry, then put in freezing conditions (−20 °C), lyophilized, ground with a mortar and pestle and stored at deep freezing conditions (−80 °C) until further analyses.

### 3.2. Nutritional Value

Spinach samples were analysed in terms of fats, ash, proteins, carbohydrates, and energetic value, according to AOAC procedures [75]. The results were expressed in g/100 g of dry matter and kcal/100 g of dry matter, in the case of energy.

### 3.3. Chemical Composition

*Free sugars.* Samples (~1 g) were spiked with melezitose as internal standard (IS, 5 mg/mL), and were extracted with 40 mL of 80% aqueous ethanol at 80 °C for 90 min. The resulting suspension was centrifuged (Centurion K24OR refrigerated centrifuge, West Sussex, UK) at 15,000 *g* for 10 min. The supernatant was concentrated at 60 °C under reduced pressure and defatted three times with 10 mL of ethyl ether. After concentration at 40 °C, the solid residues were dissolved in water to a final volume of 5 mL and filtered through Whatman 0.2 μm nylon filters. Free sugars were analysed by HPLC (Knauer, Smartline system 1000) coupled to a refractive index detector (RI detector, Knauer Smartline 2300) according to the method described by Lillian Barros et al. [76]. The results were expressed in g/100 g of dry matter.

*Organic acids.* Samples (~2 g) were extracted by stirring with 25 mL of meta-phosphoric acid (25 °C at 150 rpm) for 45 min and subsequently filtered through Whatman No. 4 paper. After the extraction procedure, organic acids were analysed via ultra-fast liquid chromatography (UFLC) coupled to photodiode array detector (PDA), employing a Shimadzu 20A series UFLC (Shimadzu Cooperation), following a methodology previously described by Barros et al. [76]. The results were expressed in g/100 g of dry matter.

*Tocopherols.* A BHT solution in hexane (10 mg/mL; 100 μL) and an IS solution in hexane (tocol; 50 μg/mL; 400 μL) were added to the sample prior to the extraction procedure. Samples (∼500 mg) were subsequently homogenized with methanol (4 mL), hexane (4 mL), and saturated NaCl aqueous solution (2 mL), by vortex mixing (1 min), and centrifuged (5 min, 4000 *g*). The clear upper layer was carefully transferred to a vial, and the procedure was repeated twice with hexane. The combined extracts were taken to dryness under nitrogen stream, redissolved in 2 mL of n-hexane, and filtered through Whatman 0.2 μm nylon filters. Tocopherols were analyzed in a Knauer Smartline system 1000 (HPLC, Berlin, Germany) coupled to a fluorescence detector (FP-2020; Jasco, Easton, MD, USA) using the internal standard methodology, as described by Pereira et al. [77]. The results were expressed in mg/100 g of dry matter.

*Fatty acids.* Following Soxhlet fat extraction, fatty acids were methylated with 5 mL of methanol:sulphuric acid:toluene 2:1:1 (*v*:*v*:*v*), during at least 12 h in a bath at 50 °C and 160 rpm; then 3 mL of deionized water were added, to obtain phase separation; the FAME were recovered with 3 mL of diethyl ether by shaking in vortex, and the upper phase was passed through a micro-column of sodium sulphate anhydrous to eliminate water residues. The sample was recovered in a vial with Teflon and filtered with Whatman 0.2 μm nylon filter. These compounds were, then, assessed by gas chromatography coupled with a flame ionization detector (GC-FID/capillary column, DANI model GC 1000, Contone, Switzerland), a split/splitless injector and a Macherey–Nagel column [76]. Results were expressed in relative percentage of each fatty acid.

### 3.4. Extracts Preparation

The samples were subjected to hydroethanolic extractions (80% ethanol) for phenolic compounds and bioactive properties evaluation. Briefly, 1 g of sample was stirred with 30 mL of hydroethanolic solution. The mixture was filtered, and the residue was re-extracted with additional 30 mL of hydroethanolic solution. After filtration, the extracts were combined and ethanol was evaporated under reduced pressure at 35 °C (rotary evaporator Büchi R-210, Flawil, Switzerland). The extracts were, then, frozen at −20 °C and lyophilized (FreeZone 4.5, Labconco, Kansas City, MO, USA) for further analysis. 

### 3.5. Phenolic Profile

The lyophilized extracts were dissolved in an ethanol:water mixture (80:20, *v*/*v*) at a concentration of 5 mg/mL and filtered (0.2 µm disposable LC filter disk, 30 mm, nylon). Afterwards, the phenolic profile was characterized by liquid chromatography with a diode-array detector (280, 330, and 370 nm wavelengths) coupled to an electrospray ionization mass spectrometry operating in negative mode (Dionex Ultimate 3000 UPLC and Linear Ion Trap LTQ XL, Thermo Scientific, San Jose, CA, USA), as previously described by the authors [78]. The phenolic compounds were identified according to their chromatographic characteristics and by comparison with the ones described in the literature. 7-levels calibration curves of appropriate standards were obtained in the range of 800–25 µg/mL for the quantitative analysis. The results were expressed in mg/g of dry weight (mg/g dw).

### 3.6. Bioactive Properties

Antioxidant activity. The lipid peroxidation inhibition and the anti-hemolytic activity of the extracts were evaluated by the decrease in thiobarbituric acid reactive substances (TBARS) and the oxidative hemolysis inhibition assay (OxHLIA), as previously described by Pereira et al. [79] and Lockowandt et al. [80], respectively. The results were expressed in IC_50_ values (sample concentration providing 50% of antioxidant activity, µg/mL). Trolox was used as positive control for both assays.

Cytotoxic activity. The cytotoxicity was determined using four tumor cell lines and a non-tumor primary culture of porcine liver cells. The lyophilized extracts were dissolved in water (8 mg/mL) for analysis, by the sulforhodamine B assay, according to Corrêa et al. [81]. The results were expressed as GI_50_ values (sample concentration that inhibited 50% of the net cell growth, in μg/mL) and Ellipticin was used as positive control.

Anti-inflammatory activity. For this analysis, the lyophilized extracts were dissolved in water (8 mg/mL) and the LPS-induced nitric oxide (NO) production was determined using the Griess Reagent System kit, with Dexamethasone as positive control. The results were expressed as EC_50_ values (μg/mL), corresponding to the sample concentration providing 50% of NO production inhibition [81].

Antimicrobial activity. Antibacterial activity was evaluated according to a previously described methodology using Gram (+) and Gram (–) bacteria [82]. The minimum inhibitory (MIC) and minimum bactericidal (MBC) concentrations were determined and streptomycin and ampicillin were used as positive controls. On the other hand, the antifungal activity was evaluated following the protocol described by Soković et al. [83]. The MIC and minimum fungicidal concentrations (MFC) were determined (mg/mL). Streptomycin and ampicillin were used as positive antibacterial controls, while ketokonazole and bifonazole were used as positive antifungal controls.

### 3.7. Statistical Analysis

The experimental design was laid out according to the split plot arrangement with main plot consisting of water stress treatments (W+ or W–), while sub-plots consisted of randomized complete blocks of the biostimulants treatments (n = 3). For the chemical analyses, three batch samples of fresh leaves harvested from all the treatments were collected. For each assay three replications were performed for each batch sample. The data were treated with the statistical package Statgraphics 5.1.plus (Statpoint Technologies, Inc., VA, USA). Means were compared with a three-way analysis of variance (two-way ANOVA) for the tested factors and their interactions when significant differences were observed the means were compared with Tukey’s honestly significant difference (HSD) test (*p* = 0.05). Moreover, the main effects of each tested factor were presented in Table 1, Table 2, Table 3 and Table 4 to show the specific trends of the individual factors.

## 4. Conclusions

The application of different biostimulant products and irrigation regimes had a variable effect on the chemical composition and bioactive properties of the tested spinach genotypes. In general, Fuji genotype presented higher fat, carbohydrates, and energy content than Viroflay genotype, while it also contained higher amounts of fructose, sucrose, raffinose and total sugars, malic acid, ascorbic acid, and total organic acids, and α- and total tocopherols. In contrast, Viroflay plants were abundant in protein, ash and γ-tocopherol. Water stress increased ash content and induced the biosynthesis of glucose and ascorbic acid, whereas normally irrigated plants were richer in carbohydrates, sucrose, raffinose, malic acid, α- and total tocopherols. Regarding the single effects of biostimulants, in most cases the tested products did not differ or presented lower values from the control treatment, except for the case of ash and glucose where Twin-Antistress and Aminovert had a profound effect, respectively. Moreover, the same treatments reduced the oxalic acid content which is considered as an antinutritional factor, while α- and total tocopherols increased by the application of Megafol and Veramin Ca. The combinatory effect of biostimulants and different irrigation regimes showed variable results depending on the genotype, especially when the water-holding regime was applied where Fuji plants showed a better response to the tested treatments since most of the parameters increased. In particular, α-and total tocopherols content increased by Megafol and Veramin Ca under water stress conditions, while α-linolenic acid content retained in similar to control treatment levels (normal irrigation and no biostimulant added) when Aminovert and Veramin Ca biostimulants were applied on water-stressed plants. For the rest of the parameters and under the water-holding regime, the application of Megafol increased fat, energy, total organic acids, Veramin Ca increased carbohydrates and increased antioxidant activity when assayed with the TBARS method, and Twin-Antistress increased raffinose and lowered oxalic acid content. In contrast, the application of biostimulants on normally irrigated plants was beneficial only in the case of Aminovert where fructose, glucose, total sugars and γ-tocopherol content increased and antioxidant activity tested with the OxHLIA assay showed the best results. Regarding the Viroflay plants, the application of Aminovert on water-stressed plants resulted in the highest protein content, whereas under normal irrigation conditions the same treatment increased total phenolic acids and total phenolic compounds content, Megafol increased saturated and monounsaturated fatty acids content and Veramin Ca improved ash content. Finally, linolenic acid was the highest under variable conditions (e.g., Aminovert application regardless of the irrigation regime or Veramin Ca application on normally irrigated plants). For, the cytotoxic activity, the tested treatments did not show promising results when compared with the positive controls. Similarly, regarding the antibacterial activity a variable effect on the tested bacteria was recorded depending on the tested biostimulant, irrigation regime and genotype, whereas none of the tested samples showed promising antifungal activity since MIC values were considerably higher than the positive controls. In conclusion, a variable effect of the tested biostimulants and irrigation regimes was observed on bioactive properties and chemical composition of both spinach genotypes which highlights the need for further research in order to make profound conclusions regarding the positive effects of biostimulants under water stress conditions.

## Figures and Tables

**Table 1:** Proximate composition (g/100 g dw) and energetic value (kcal/100 g dw) of spinach leaves in relation to genotype, biostimulant, and irrigation treatment, and their combinatorial effect.

	Treatment	Fat	Protein	Ash	Carbohydrates	Energy
**Genotype**	Fuji	7.8 ± 1.6a	35.8 ± 1.8b	18.55 ± 1.02b	37.8 ± 2.6a	364.9 ± 10.3a
	Viroflay	6.2 ± 1.7b	38.1 ± 1.7a	21.4 ± 1.7a	34.2 ± 1.5b	345.5 ± 11.1b
**Water stress**	W–	6.9 ± 2.0	36.5 ± 2.7	19.4 ± 1.3b	37.2 ± 2.8a	356.9 ± 13.7
	W+	7.12 ± 1.64	37 ± 1	20.5 ± 2.3a	34.9 ± 2.2b	353 ± 15
**Biostimulant**	Control	7.3 ± 0.7a	38.2 ± 0.9a	19.8 ± 1.9b	35 ± 2	357.2 ± 10.8a
	AM	6.78 ± 0.9a	38 ± 3a	19.4 ± 1.6b	36.1 ± 2.8	356.1 ± 10.8a
	MEG	7.7 ± 2.1a	36.8 ± 1.9a	19.5 ± 1.2b	35.9 ± 1.2	360 ± 15a
	TA	5.3 ± 1.6b	37 ± 1a	21.5 ± 2.6a	36 ± 3	340.3 ± 15.9b
	V	8 ± 1a	35.2 ± 1.8b	19.6 ± 1.8b	37.0 ± 3.9	361.8 ± 7.3a
**Fuji**	CW–	7.2 ± 0.3e	37.2 ± 0.6fg	18.97 ± 0.06gh	36.6 ± 0.1e	360 ± 1de
	AMW–	9.4 ± 0.3b	33.93 ± 0.05l	18.9 ± 0.6hi	37.8 ± 0.3d	371 ± 3b
	MEGW–	10.7 ± 0.3a	33.8 ± 0.4l	17.53 ± 0.02jk	37.8 ± 0.2d	383.3 ± 0.9a
	TAW–	4.4 ± 0.1i	35.4 ± 0.1k	19.3 ± 0.1fg	40.97 ± 0.05b	344.6 ± 0.8j
	VW–	7.3 ± 0.4e	32 ± 1m	17.4 ± 0.3jk	42.9 ± 0.3a	367.0 ± 0.3c
	CW+	8.3 ± 0.4c	38 ± 1ef	17.3 ± 0.4k	36.5 ± 0.7e	372 ± 2b
	AMW+	5.9 ± 0.2g	36.9 ± 0.3gh	17.7 ± 0.6j	39.4 ± 0.1c	359 ± 2ef
	MEGW+	8.0 ± 0.3d	37.0 ± 0.2gh	20.4 ± 0.1d	34.68 ± 0.06h	358 ± 1ef
	TAW+	7.9 ± 0.3d	38.05 ± 0.03de	19.5 ± 0.2f	34.6 ± 0.3h	361.6 ± 0.6d
	VW+	9.1 ± 0.4b	35.71 ± 0.04jk	18.6 ± 0.2i	36.6 ± 0.4e	371.1 ± 0.9b
**Viroflay**	CW–	6.8 ± 0.3f	39.2 ± 0.6b	20.6 ± 0.5d	33.5 ± 0.1i	351.5 ± 0.4i
	AMW–	4.3 ± 0.2i	42.0 ± 0.3a	19.3 ± 0.2fg	34.4 ± 0.2h	344 ± 1j
	MEGW–	7.2 ± 0.3e	37.6 ± 0.5ef	19.84 ± 0.08e	35.3 ± 0.2g	357 ± 1fg
	TAW–	4.8 ± 0.2h	36.6 ± 0.2hi	21.983 ± 0.001bc	36.7 ± 0.2e	335.9 ± 0.5k
	VW–	7.2 ± 0.2e	37 ± 1hi	20.5 ± 0.5d	35.8 ± 0.5fg	354 ± 2h
	CW+	6.8 ± 0.3f	38.4 ± 0.2cd	22.33 ± 0.03b	32.4 ± 0.3j	345 ± 1j
	AMW+	7.5 ± 0.2e	38.0 ± 0.7de	21.9 ± 0.2c	32.6 ± 0.5j	350 ± 1i
	MEGW+	5.0 ± 0.2h	38.8 ± 0.4bc	20.3 ± 0.3d	35.9 ± 0.3f	344 ± 2j
	TAW+	4.2 ± 0.2i	37.2 ± 0.1fg	25.4 ± 0.1a	33.30 ± 0.05i	319.4 ± 0.9l
	VW+	8.5 ± 0.2c	36.3 ± 0.1ij	21.9 ± 0.5c	32.7 ± 0.4j	358 ± 3gh

F values: F_Genotype_: 18.5; F_Water-stress_: 7.71; F_Biostimulant_: 2.79; F_Interaction_: 2.79. Degrees of freedom (DF): DF_Genotype_: 1; DF_Water-stress_: 1; DF_Biostimulant_: 4; DF_Interaction_: 4. ^¥^W+: indicates normal irrigation regime; W–: indicates water-holding irrigation regime; C: Control; AM: Aminovert; MEG: Megafol; TA: Twin-Antistress; V: Veramin Ca. Means in the same column and for the same genotype, irrigation, and biostimulant treatment and their interactions followed by different Latin letters are significantly different according to Tukey’s HSD test (*p* = 0.05).

**Table 2:** Free sugars content (g/100 g dw) of spinach leaves in relation to genotype, biostimulant, and irrigation treatment.

	Treatment	Fructose	Glucose	Sucrose	Raffinose	Total
**Genotype**	Fuji	1.42 ± 0.03a	2.46 ± 0.05	2.056 ± 0.03a	2.736 ± 0.08a	8.66 ± 0.09a
	Viroflay	0.43 ± 0.01b	2.11 ± 0.03	0.86 ± 0.02b	2.266 ± 0.005b	5.68 ± 0.06b
**Water stress**	W–	0.965 ± 0.003	2.06 ± 0.06b	1.71 ± 0.06a	2.743 ± 0.001a	7.488 ± 0.006
	W+	0.887 ± 0.002	2.51 ± 0.03a	1.21 ± 0.02b	2.26 ± 0.07b	6.86 ± 0.03
**Biostimulant**	Control	0.94 ± 0.05	2.148 ± 0.003ab	1.75 ± 0.03	2.79 ± 0.08a	7.63 ± 0.05
	AM	1.07 ± 0.03	2.58 ± 0.03a	1.57 ± 0.06	2.67 ± 0.02a	7.89 ± 0.06
	MEG	0.89 ± 0.07	2.22 ± 0.08ab	1.22 ± 0.04	2.41 ± 0.06ab	6.73 ± 0.08
	TA	0.79 ± 0.03	2.66 ± 0.03a	1.22 ± 0.03	2.17 ± 0.04b	6.83 ± 0.03
	V	0.94 ± 0.06	1.8 ± 0.3b	1.55 ± 0.08	2.47 ± 0.01ab	6.76 ± 0.01
**Fuji**	CW–	1.28 ± 0.02f	2.05 ± 0.09i	2.8 ± 0.1a	3.274 ± 0.007ab	9.373 ± 0.001cd
	AMW–	1.47 ± 0.01d	1.75 ± 0.02l	2.266 ± 0.005cd	2.69 ± 0.03e	8.17 ± 0.05f
	MEGW–	1.60 ± 0.08b	2.6 ± 0.1fg	2.14 ± 0.06e	3.10 ± 0.03c	9.4 ± 0.2bc
	TAW–	1.25 ± 0.03f	2.431 ± 0.003h	2.22 ± 0.02d	3.34 ± 0.08a	9.24 ± 0.03de
	VW–	1.37 ± 0.02e	2.75 ± 0.03e	2.41 ± 0.07b	2.53 ± 0.06f	9.1 ± 0.2e
	CW+	1.55 ± 0.06c	3.2 ± 0.2c	2.43 ± 0.09b	2.5 ± 0.1fg	9.6 ± 0.3b
	AMW+	2.1 ± 0.1a	3.43 ± 0.02a	2.3 ± 0.1c	2.86 ± 0.02d	10.74 ± 0.02a
	MEGW+	1.117 ± 0.005g	2.5 ± 0.1gh	0.89 ± 0.03j	2.4 ± 0.1h	6.9 ± 0.3h
	TAW+	1.02 ± 0.03h	1.92 ± 0.08jk	1.86 ± 0.07f	2.3 ± 0.1i	7.1 ± 0.3h
	VW+	1.42 ± 0.06d	2.0 ± 0.1ij	1.24 ± 0.03h	2.4 ± 0.1gh	7.0 ± 0.1h
**Viroflay**	CW–	0.71 ± 0.02i	0.84 ± 0.03m	1.26 ± 0.05h	3.05 ± 0.04c	5.86 ± 0.09j
	AMW–	0.47 ± 0.02j	2.62 ± 0.05f	1.00 ± 0.01i	3.21 ± 0.02b	7.298 ± 0.004g
	MEGW–	0.423 ± 0.009jkl	1.83 ± 0.06kl	0.95 ± 0.01ij	1.75 ± 0.02l	4.96 ± 0.05m
	TAW–	0.41 ± 0.02l	2.99 ± 0.08d	0.35 ± 0.01o	1.56 ± 0.05m	5.3 ± 0.2l
	VW–	0.675 ± 0.004i	0.72 ± 0.03n	1.77 ± 0.05g	2.91 ± 0.02d	6.08 ± 0.09i
	CW+	0.244 ± 0.002n	2.536 ± 0.005fg	0.54 ± 0.02m	2.36 ± 0.07hi	5.68 ± 0.05jk
	AMW+	0.222 ± 0.003n	2.52 ± 0.02fgh	0.683 ± 0.004l	1.93 ± 0.06k	5.36 ± 0.08l
	MEGW+	0.42 ± 0.01kl	1.94 ± 0.02j	0.89 ± 0.02j	2.386 ± 0.003h	5.64 ± 0.02k
	TAW+	0.466 ± 0.006jk	3.32 ± 0.04b	0.45 ± 0.02n	1.48 ± 0.04n	5.71 ± 0.01jk
	VW+	0.299 ± 0.003m	1.77 ± 0.02l	0.789 ± 0.001k	2.02 ± 0.07j	4.87 ± 0.09m

F values: F_Genotype_: 18.5; F_Water-stress_: 7.71; F_Biostimulant_: 2.79; F_Interaction_: 2.79. Degrees of freedom (DF): DF_Genotype_: 1; DF_Water-stress_: 1; DF_Biostimulant_: 4; DF_Interaction_: 4. ^¥^W+: indicates normal irrigation regime; W–: indicates water-holding irrigation regime; C: Control; AM: Aminovert; MEG: Megafol; TA: Twin-Antistress; V: Veramin Ca. Means in the same column and for the same genotype, irrigation, and biostimulant treatment and their interactions followed by different Latin letters are significantly different according to Tukey’s HSD test (*p* = 0.05).

**Table 3:** Organic acids content (g/100 g dw) of spinach leaves in relation to genotype, biostimulant and irrigation treatment.

8	Treatment	Oxalic Acid	Malic Acid	Fumaric Acid	Ascorbic Acid	Total
**Genotype**	Fuji	4.20 ± 0.03	2.951 ± 0.007a	tr	0.0097 ± 0.0001a	7.158 ± 0.008a
	Viroflay	4.41 ± 0.06	2.24 ± 0.06b	tr	0.0040 ± 0.0001b	6.65 ± 0.06b
**Water stress**	W–	4.20 ± 0.06	2.803 ± 0.005a	tr	0.0055 ± 0.002b	7.008 ± 0.002
	W+	4.41 ± 0.05	2.39 ± 0.04b	tr	0.0108 ± 0.0001a	6.8 ± 0.1
**Biostimulant**	Control	4.45 ± 0.05a	2.72 ± 0.06	tr	0.0135 ± 0.0003a	7.17 ± 0.03a
	AM	4.04 ± 0.01b	2.68 ± 0.02	tr	0.0103 ± 0.0001ab	6.72 ± 0.01ab
	MEG	4.44 ± 0.03a	2.58 ± 0.03	tr	0.0015 ± 0.0002c	7.02 ± 0.03ab
	TA	4.15 ± 0.08ab	2.46 ± 0.04	tr	0.0092 ± 0.0006ab	6.61 ± 0.05b
	V	4.446 ± 0.005a	2.54 ± 0.02	tr	0.0081 ± 0.001b	6.99 ± 0.01ab
**Fuji**	CW–	4.3 ± 0.1ef	3.1 ± 0.1c	tr	0.0087 ± 0.0001cd	7.41 ± 0.01c
	AMW–	3.76 ± 0.03h	3.6 ± 0.1a	tr	n.d.	7.4 ± 0.1c
	MEGW–	4.7 ± 0.1b	3.27 ± 0.07b	tr	0.0008 ± 0.0001e	8.0 ± 0.2a
	TAW–	3.3 ± 0.1j	3.0 ± 0.1c	tr	n.d.	6.27 ± 0.03jk
	VW–	4.5 ± 0.2cd	3.30 ± 0.08b	tr	0.0067 ± 0.0002cd	7.78 ± 0.09b
	CW+	4.32 ± 0.06ef	2.77 ± 0.06de	tr	0.0183 ± 0.0007a	7.108 ± 0.002de
	AMW+	4.5 ± 0.2cd	2.7 ± 0.1ef	tr	0.0148 ± 0.0004b	7.2 ± 0.3d
	MEGW+	4.4 ± 0.1de	2.575 ± 0.004g	tr	n.d.	7.0 ± 0.1ef
	TAW+	3.6 ± 0.1i	2.5 ± 0.1gh	tr	0.0092 ± 0.0004c	6.1 ± 0.2l
	VW+	4.7 ± 0.1b	2.74 ± 0.04de	tr	0.0095 ± 0.0001c	7.4 ± 0.2c
**Viroflay**	CW–	4.5 ± 0.1cd	2.20 ± 0.04i	tr	n.d.	6.66 ± 0.08i
	AMW–	4.3 ± 0.1ef	2.5 ± 0.1gh	tr	0.0057 ± 0.0002d	6.8 ± 0.2hi
	MEGW–	4.0 ± 0.2g	2.2 ± 0.1i	tr	n.d.	6.2 ± 0.1kl
	TAW–	4.6 ± 0.2bc	2.61 ± 0.04fg	tr	n.d.	7.2 ± 0.2d
	VW–	4.2 ± 0.1f	2.23 ± 0.05i	tr	n.d.	6.44 ± 0.07j
	CW+	4.7 ± 0.2b	2.8 ± 0.1d	tr	n.d.	7.5 ± 0.4c
	AMW+	3.7 ± 0.2hi	1.89 ± 0.04j	tr	n.d.	5.6 ± 0.1m
	MEGW+	4.66 ± 0.02b	2.28 ± 0.07i	tr	0.0022 ± 0.0001e	6.9 ± 0.1fg
	TAW+	5.15 ± 0.02a	1.73 ± 0.02k	tr	n.d.	6.88 ± 0.04gh
	VW+	4.40 ± 0.04de	1.91 ± 0.01j	tr	n.d.	6.31 ± 0.0jk

F values: F_Genotype_: 18.5; F_Water-stress_: 7.71; F_Biostimulant_: 2.79; F_Interaction_: 2.79. Degrees of freedom (DF): DF_Genotype_: 1; DF_Water-stress_: 1; DF_Biostimulant_: 4; DF_Interaction_: 4. ^¥^W+: indicates normal irrigation regime; W–: indicates water-holding irrigation regime; C: Control; AM: Aminovert; MEG: Megafol; TA: Twin-Antistress; V: Veramin Ca. Means in the same column and for the same genotype, irrigation, and biostimulant treatment and their interactions followed by different Latin letters are significantly different according to Tukey’s HSD test (*p* = 0.05).

**Table 4:** Tocopherols content (mg/100 g dw) of spinach leaves in relation to genotype, biostimulant and irrigation treatment.

	Treatment	α-Tocopherol	γ-Tocopherol	Total
**Genotype**	Fuji	8.01 ± 0.09a	0.763 ± 0.008b	8.77 ± 0.07a
	Viroflay	6.55 ± 0.03b	0.989 ± 0.003a	7.539 ± 0.002b
**Water stress**	W–	8.117 ± 0.005a	0.86 ± 0.06	8.975 ± 0.003a
	W+	6.44 ± 0.08b	0.89 ± 0.01	7.335 ± 0.008b
**Biostimulant**	Control	6.39 ± 0.02bc	0.86 ± 0.01a	7.26 ± 0.03b
	AM	7.44 ± 0.01ab	1.03 ± 0.02a	8.48 ± 0.02a
	MEG	8.46 ± 0.08a	0.89 ± 0.06a	9.35 ± 0.02a
	TA	5.51 ± 0.02c	0.68 ± 0.02b	6.19 ± 0.06c
	V	8.59 ± 0.03a	0.91 ± 0.03a	9.49 ± 0.04a
**Fuji**	CW–	5.2 ± 0.1j	0.60 ± 0.02kl	5.8 ± 0.1m
	AMW–	9.9 ± 0.2b	0.63 ± 0.03jk	10.6 ± 0.2b
	MEGW–	10.5 ± 0.4a	0.69 ± 0.02hi	11.2 ± 0.3a
	TAW–	6.72 ± 0.04g	0.85 ± 0.02ef	7.57 ± 0.06gh
	VW–	10.8 ± 0.2a	0.70 ± 0.03hi	11.5 ± 0.2a
	CW+	6.685 ± 0.008g	0.762 ± 0.008g	7.446 ± 0.001hi
	AMW+	7.03 ± 0.05f	1.30 ± 0.05a	8.338 ± 0.001f
	MEGW+	9.2 ± 0.5c	0.84 ± 0.03f	10.0 ± 0.5c
	TAW+	5.6 ± 0.1i	0.60 ± 0.01kl	6.2 ± 0.1l
	VW+	8.4 ± 0.3d	0.66 ± 0.03ij	9.1 ± 0.4e
**Viroflay**	CW–	8.07 ± 0.02e	1.21 ± 0.03b	9.28 ± 0.05de
	AMW–	6.71 ± 0.07g	1.09 ± 0.02d	7.81 ± 0.09g
	MEGW–	8.3 ± 0.2de	1.15 ± 0.05c	9.4 ± 0.3d
	TAW–	6.531 ± 0.008g	0.58 ± 0.02l	7.11 ± 0.02j
	VW–	8.4 ± 0.2d	1.07 ± 0.03d	9.5 ± 0.2d
	CW+	5.67 ± 0.02i	0.882 ± 0.005e	6.55 ± 0.01k
	AMW+	6.09 ± 0.04h	1.11 ± 0.02cd	7.20 ± 0.02ij
	MEGW+	5.9 ± 0.1hi	0.889 ± 0.006e	6.7 ± 0.2k
	TAW+	3.21 ± 0.02k	0.705 ± 0.003h	3.92 ± 0.02n
	VW+	6.65 ± 0.04g	1.20 ± 0.02b	7.85 ± 0.01g

F values: F_Genotype_: 18.5; F_Water-stress_: 7.71; F_Biostimulant_: 2.79; F_Interaction_: 2.79. Degrees of freedom (DF): DF_Genotype_: 1; DF_Water-stress_: 1; DF_Biostimulant_: 4; DF_Interaction_: 4. ^¥^W+: indicates normal irrigation regime; W–: indicates water-holding irrigation regime; C: Control; AM: Aminovert; MEG: Megafol; TA: Twin-Antistress; V: Veramin Ca. Means in the same column and for the same genotype, irrigation, and biostimulant treatment and their interactions followed by different Latin letters are significantly different according to Tukey’s HSD test (*p* = 0.05).

**Table 5:** Fatty acids content (relative %) of spinach leaves in relation to genotype, biostimulant and irrigation treatment.

	Treatment	C6:0	C8:0	C10:0	C12:0	C14:0	C15:0	C16:0	C16:1	C17:0	C18:0	C18:1n9c	C18:2n6c	C18:3n6	C18:3n3	C20:0
**Fuji**	CW–	nd	nd	0.027 ± 0.001fg	0.038 ± 0.003e	0.15 ± 0.01fg	0.101 ± 0.006f	8.8 ± 0.1c	1.68 ± 0.05a	0.066 ± 0.003c	0.95 ± 0.06c	2.19 ± 0.05def	12.1 ± 0.2ij	nd	72.37 ± 0.04d	0.17 ± 0.01j
	AMW–	nd	nd	0.037 ± 0.003d	0.023 ± 0.001i	0.111 ± 0.001kl	0.057 ± 0.001k	7.0 ± 0.3k	1.00 ± 0.04j	0.041 ± 0.001m	0.74 ± 0.05fgh	1.9 ± 0.1k	11.7 ± 0.3j	nd	75.7 ± 0.2a	0.162 ± 0.009jk
	MEGW–	0.028 ± 0.002b	0.016 ± 0.001d	0.028 ± 0.001f	0.03 ± 0.01gh	0.133 ± 0.001j	0.086 ± 0.001h	7.7 ± 0.3g	1.4 ± 0.1d	0.061 ± 0.003d	0.91 ± 0.06c	2.57 ± 0.06b	14.7 ± 0.4c	0.17 ± 0.01	71±2f	0.199 ± 0.006h
	TAW–	nd	0.011 ± 0.001f	0.026 ± 0.001g	0.028 ± 0.001h	0.111 ± 0.009kl	0.078 ± 0.002j	7.6 ± 0.7gh	1.327 ± 0.003ef	0.055 ± 0.001ef	0.698 ± 0.008hi	2.27 ± 0.08cd	13.1 ± 0.7ef	nd	73±2c	0.157 ± 0.006jk
	VW–	0.023 ± 0.001c	0.007 ± 0.001g	0.013 ± 0.001kl	0.045 ± 0.001d	0.14 ± 0.01hij	0.091 ± 0.004g	7.7 ± 0.1gh	1.26 ± 0.04fgh	0.054 ± 0.001fg	0.62 ± 0.01kl	2.1 ± 0.1ghi	12.20 ± 0.08hij	nd	75.1 ± 0.3a	0.177 ± 0.004i
	CW+	nd	nd	0.013 ± 0.001kl	0.017 ± 0.001k	0.118 ± 0.006k	0.083 ± 0.001hi	7.31 ± 0.04ijk	1.54 ± 0.04c	0.051 ± 0.001hij	0.71 ± 0.02ghi	1.951 ± 0.006jk	12.5 ± 0.2ghi	nd	75.2 ± 0.2a	0.163 ± 0.006jk
	AMW+	nd	nd	0.011 ± 0.001m	0.022 ± 0.001ij	0.099 ± 0.006m	0.079 ± 0.001ij	7.6 ± 0.5ghi	1.33 ± 0.04e	0.061 ± 0.001d	0.64 ± 0.02jk	2.1 ± 0.1gh	12.4 ± 0.2ghi	nd	74 ± 1b	0.155 ± 0.003k
	MEGW+	nd	nd	0.023 ± 0.001h	0.037 ± 0.001e	0.143 ± 0.009hi	0.098 ± 0.002f	7.6 ± 0.7hij	1.142 ± 0.003i	0.062 ± 0.004d	0.795 ± 0.008e	1.96 ± 0.08ijk	14.9 ± 0.7bc	nd	71.6 ± 0.6e	0.23 ± 0.02ef
	TAW+	nd	nd	0.012 ± 0.001lm	0.019 ± 0.001jk	0.91 ± 0.002m	0.077 ± 0.001j	7.2 ± 0.3jk	1.46 ± 0.05d	0.049 ± 0.003jk	0.60 ± 0.03kl	2.03 ± 0.02ghi	12.6 ± 0.2fgh	nd	74 ± 1b	0.134 ± 0.002l
	VW+	0.03 ± 0.01a	0.014 ± 0.001e	0.034 ± 0.001e	0.066 ± 0.004c	0.28 ± 0.01c	0.121 ± 0.008d	8.2 ± 0.1ef	1.27 ± 0.05efg	0.067 ± 0.004bc	0.68 ± 0.02ij	1.67 ± 0.03l	12.5 ± 0.3ghi	nd	73.6 ± 0.2bc	0.23 ± 0.02f
**Viroflay**	CW–	nd	0.026 ± 0.001c	0.054 ± 0.001c	0.092 ± 0.006b	0.242 ± 0.003d	0.115 ± 0.008e	8.6 ± 0.3cd	1.2 ± 0.1gh	0.057 ± 0.001e	1.04 ± 0.02b	2.23 ± 0.02de	12.8 ± 0.1fg	nd	71.2 ± 0.5ef	0.243 ± 0.004cd
	AMW–	nd	nd	0.014 ± 0.001jk	0.02 ± 0.001ijk	0.111 ± 0.001l	0.097 ± 0.005f	8.1 ± 0.1f	1.60 ± 0.09bc	0.045 ± 0.002l	0.58 ± 0.02l	2.34 ± 0.02c	16 ± 1a	nd	69 ± 1g	0.232 ± 0.001ef
	MEGW–	nd	nd	0.015 ± 0.001j	0.037 ± 0.001e	0.16 ± 0.01f	0.092 ± 0.006g	8.1 ± 0.1f	1.44 ± 0.04d	0.044 ± 0.004l	0.76 ± 0.06efg	2.03 ± 0.01hij	13.40 ± 0.02de	nd	71.6 ± 0.3e	0.292 ± 0.006ab
	TAW–	nd	nd	0.025 ± 0.001g	0.046 ± 0.001d	0.245 ± 0.001d	0.133 ± 0.004c	9.20 ± 0.09b	1.46 ± 0.04d	0.052 ± 0.004ghi	0.93 ± 0.07c	2.18 ± 0.03def	14.74 ± 0.02bc	nd	68.5 ± 0.3h	0.29 ± 0.01a
	VW–	nd	nd	0.014 ± 0.001jkl	0.036 ± 0.001ef	0.15 ± 0.01gh	0.113 ± 0.004e	8.48 ± 0.04de	1.400 ± 0.001d	0.049 ± 0.001ijk	0.78 ± 0.05ef	2.10 ± 0.04ghi	13.91 ± 0.03d	nd	70.7 ± 0.2f	0.284 ± 0.005b
	CW+	nd	0.032 ± 0.002b	0.069 ± 0.005b	0.091 ± 0.005b	0.321 ± 0.004b	0.138 ± 0.004b	9.2 ± 0.3b	1.59 ± 0.06bc	0.069 ± 0.001b	0.93 ± 0.02c	2.20 ± 0.04def	13.82 ± 0.03d	nd	69.0 ± 0.1gh	0.30 ± 0.01a
	AMW+	nd	nd	0.018 ± 0.001i	0.035 ± 0.002ef	0.139 ± 0.006hij	0.092 ± 0.005g	8.33 ± 0.01ef	1.26 ± 0.03gh	0.047 ± 0.001k	0.87 ± 0.05d	2.6 ± 0.2b	15.5 ± 0.8a	nd	68.88 ± 0.07gh	0.249 ± 0.001c
	MEGW+	nd	0.033 ± 0.001a	0.111 ± 0.001a	0.132 ± 0.008a	0.59 ± 0.01a	0.154 ± 0.005a	9.57 ± 0.04a	1.60 ± 0.09bc	0.082 ± 0.001a	1.282 ± 0.001a	3.086 ± 0.006a	14.622 ± 0.004c	nd	66.44 ± 0.07i	0.245 ± 0.001cd
	TAW+	nd	nd	0.027 ± 0.001fg	0.033 ± 0.001fg	0.173 ± 0.004e	0.117 ± 0.001de	8.46 ± 0.03de	1.20 ± 0.04hi	0.052 ± 0.001ghi	0.640 ± 0.001jk	2.15 ± 0.02fgh	15.3 ± 0.2ab	nd	68.9 ± 0.2gh	0.218 ± 0.009g
	VW+	nd	nd	0.037 ± 0.001d	0.031 ± 0.001gh	0.135 ± 0.006ij	0.098 ± 0.003f	9.16 ± 0.16b	1.61 ± 0.02bc	0.053 ± 0.001gh	0.86 ± 0.05d	2.13 ± 0.04gh	14.8 ± 0.03bc	nd	68.8 ± 0.06gh	0.239 ± 0.004de

F values: F_Genotype_: 18.5; F_Water-stress_: 7.71; F_Biostimulant_: 2.79; F_Interaction_: 2.79. Degrees of freedom (DF): DF_Genotype_: 1; DF_Water-stress_: 1; DF_Biostimulant_: 4; DF_Interaction_: 4. ^¥^W+: indicates normal irrigation regime; W–: indicates water-holding irrigation regime; C: Control; AM: Aminovert; MEG: Megafol; TA: Twin-Antistress; V: Veramin Ca. Means in the same column followed by different Latin letters are significantly different according to Tukey’s HSD test (*p* = 0.05). Caproic acid (C6:0); Caprylic acid (C8:0); Capric acid (C10:0); Lauric acid (C12:0); Myristic acid (C14:0); Pentadecylic acid (C15:0); Palmitic acid (C16:0); Palmitoleic acid (C16:1); Margaric acid (C17:0); Stearic acid (C18:0); Oleic acid (C18:1n9); Linoleic acid (C18:2n6c); γ-Linolenic acid (C18:3n6); α-Linolenic acid (C18:3n3); Arachidic acid (C20:0).

**Table 6:** Fatty acids content (relative %) of spinach leaves in relation to genotype, biostimulant and irrigation treatment.

	Treatment	C20:1	C20:2	C20:3n6	C20:3n3	C20:4n6	C20:5n3	C21:0	C22:0	C22:6n3	C23:0	C24:0	SFA	MUFA	PUFA
**Fuji**	CW–	0.167 ± 0.001gh	0.32 ± 0.006a	0.55 ± 0.01a	nd	nd	nd	0.137 ± 0.003a	0.124 ± 0.004k	nd	nd	0.083 ± 0.005h	10.6 ± 0.2fg	4.027 ± 0.002cd	85.3 ± 0.2fg
	AMW–	0.137 ± 0.008k	0.070 ± 0.004m	0.152 ± 0.009c	nd	nd	0.052 ± 0.002k	nd	0.193 ± 0.001j	0.218 ± 0.006a	nd	0.68 ± 0.04f	9.1 ± 0.4k	3.0 ± 0.2j	87.9 ± 0.2a
	MEGW–	0.155 ± 0.001ij	0.1159 ± 0.001jk	nd	0.128 ± 0.004f	nd	0.070 ± 0.002h	nd	0.231 ± 0.009h	nd	0.050 ± 0.001hi	0.61 ± 0.05g	10.1 ± 0.3hi	4.12 ± 0.05bc	85.8 ± 0.8e
	TAW–	0.148 ± 0.001j	0.115 ± 0.001k	Nd	0.106 ± 0.002g	nd	nd	nd	0.253 ± 0.009g	nd	0.047 ± 0.001i	0.70 ± 0.05f	9.8 ± 0.7ij	3.72 ± 0.07f	86.5 ± 0.8d
	VW–	0.122 ± 0.006l	nd	0.040 ± 0.002e	nd	0.153 ± 0.004b	nd	0.069 ± 0.004b	nd	nd	nd	0.045 ± 0.002i	9.0 ± 0.1k	3.5 ± 0.1h	87.5 ± 0.2bc
	CW+	0.157 ± 0.001hij	0.126 ± 0.003j	0.061 ± 0.004d	nd	nd	nd	0.009 ± 0.001d	nd	nd	nd	0.035 ± 0.002i	8.506 ± 0.009l	3.63 ± 0.03fg	87.85 ± 0.02ab
	AMW+	0.127 ± 0.006kl	0.135 ± 0.009i	0.149 ± 0.008c	nd	nd	0.058 ± 0.004j	nd	0.25 ± 0.02gh	nd	nd	0.819 ± 0.003e	9.7 ± 0.5j	3.5 ± 0.2h	86.8 ± 0.8d
	MEGW+	0.167 ± 0.002g	0.102 ± 0.004l	0.016 ± 0.001f	0.127 ± 0.008f	nd	0.048 ± 0.001l	nd	0.31 ± 0.02f	nd	nd	0.92 ± 0.04d	10.02 ± 0.03hij	3.28 ± 0.03i	86.7 ± 0.2d
	TAW+	0.163 ± 0.006ghi	0.113 ± 0.001k	nd	0.156 ± 0.002c	nd	0.062 ± 0.001i	nd	0.212 ± 0.001i	nd	nd	0.70 ± 0.04f	9.1 ± 0.7k	3.69 ± 0.07fg	87.2 ± 0.4c
	VW+	0.159 ± 0.008ghi	0.208 ± 0.008b	0.194 ± 0.008b	nd	nd	nd	0.060 ± 0.005c	0.38 ± 0.03c	0.156 ± 0.006b	nd	0.028 ± 0.001i	10.2 ± 0.1h	3.10 ± 0.03j	86.68 ± 0.08d
**Viroflay**	CW–	0.25 ± 0.02ab	0.151 ± 0.008ef	nd	0.156 ± 0.006c	nd	0.081 ± 0.001f	nd	0.357 ± 0.005de	0.035 ± 0.001e	0.073 ± 0.002ef	0.894 ± 0.001d	11.8 ± 0.4c	3.7 ± 0.1fg	84.5 ± 0.5i
	AMW–	0.22 ± 0.01f	0.173 ± 0.007c	nd	0.157 ± 0.006bc	nd	0.074 ± 0.001g	nd	0.29 ± 0.01f	0.042 ± 0.001d	0.058 ± 0.004g	0.832 ± 0.004e	10.33 ± 0.07gh	4.18 ± 0.08b	85.5 ± 0.2ef
	MEGW–	0.24 ± 0.02bc	0.143 ± 0.006gh	nd	0.161 ± 0.003b	nd	0.131 ± 0.004c	nd	0.34 ± 0.02e	0.023 ± 0.001g	0.070 ± 0.001f	0.912 ± 0.001d	10.9 ± 0.2ef	3.717 ± 0.004fg	85.43 ± 0.09ef
	TAW–	0.228 ± 0.001de	0.147 ± 0.002fg	nd	0.147 ± 0.001d	nd	0.140 ± 0.003b	nd	0.414 ± 0.003b	0.037 ± 0.001e	0.098 ± 0.007b	1.02 ± 0.05b	12.5 ± 0.2b	3.9 ± 0.1e	83.7 ± 0.3k
	VW–	0.23 ± 0.01ef	0.138 ± 0.008hi	nd	0.172 ± 0.004a	nd	0.082 ± 0.001f	nd	0.39 ± 0.02c	0.025 ± 0.001g	0.091 ± 0.001c	0.89 ± 0.05d	11.28 ± 0.03d	3.72 ± 0.06f	85.0 ± 0.3gh
	CW+	0.24 ± 0.01cd	0.155 ± 0.004de	nd	0.138 ± 0.001e	nd	0.09 ± 0.001e	nd	0.42 ± 0.02b	0.023 ± 0.001g	0.076 ± 0.006e	1.02 ± 0.03b	12.7 ± 0.4b	4.0 ± 0.1cd	83.3 ± 0.2l
	AMW+	0.251 ± 0.003a	0.136 ± 0.001hi	nd		0.17 ± 0.008a	0.117 ± 0.004d	nd	0.37 ± 0.02cd	0.036 ± 0.003e	0.069 ± 0.002f	0.90 ± 0.03d	11.11 ± 0.01de	4.1 ± 0.2bcd	84.83 ± 0.02h
	MEGW+	0.23 ± 0.01ef	0.179 ± 0.008c	nd	0.155 ± 0.001c	nd	0.081 ± 0.001f	nd	0.377 ± 0.004c	0.037 ± 0.002e	0.052 ± 0.001h	0.977 ± 0.006c	13.60 ± 0.04a	4.9 ± 0.1a	81.1 ± 0.3m
	TAW+	0.237 ± 0.008cde	0.161 ± 0.008d	nd	0.127 ± 0.004f	nd	0.169 ± 0.002a	nd	0.51 ± 0.02a	0.063 ± 0.002c	0.103 ± 0.002a	1.34 ± 0.02a	11.7 ± 0.4c	3.59 ± 0.0gh2	84.7 ± 0.2hi
	VW+	0.245 ± 0.003abc	0.150 ± 0.006efg	nd	0.145 ± 0.001d	nd	0.080 ± 0.001f	nd	0.358 ± 0.008de	0.03 ± 0.002f	0.086 ± 0.007d	0.90 ± 0.03d	11.97 ± 0.06c	3.99 ± 0.02de	84.1 ± 0.2j

F values: F_Genotype_: 18.5; F_Water-stress_: 7.71; F_Biostimulant_: 2.79; F_Interaction_: 2.79. Degrees of freedom (DF): DF_Genotype_: 1; DF_Water-stress_: 1; DF_Biostimulant_: 4; DF_Interaction_: 4. ^¥^W+: indicates normal irrigation regime; W–: indicates water-holding irrigation regime; C: Control; AM: Aminovert; MEG: Megafol; TA: Twin-Antistress; V: Veramin Ca. Means in the same column followed by different Latin letters are significantly different according to Tukey’s HSD test (*p* = 0.05). Paulinic acid (C20:1); Eicosadienoic acid (C20:2); Dihomo-γ-linolenic acid (C20:3n6); Eicosatrienoic acid (C20:3n3); Arachidonic acid (C20:4n6); Eicosapentaeonic acid (C20:5n3); Heneicosylic acid (C21:0); Behenic acid (C22:0); Docosahexaenoic acid (C22:6n3); Tricosylic acid (C23:0); Lignoceric acid (C24:0); SFA: saturated fatty acids; MUFA: monounsaturated fatty acids; PUFA: polyunsaturated fatty acids.

**Table 7:** Retention time (Rt), wavelengths of maximum absorption in the visible region (λ_max_), mass spectral data, and tentative identification of phenolic compounds of spinach leaves in relation to genotype, biostimulant and irrigation treatment.

Peak	Rt (min)	λ_max_ (nm)	[M − H]^−^ (*m/z*)	MS^2^ (*m*/*z*)	Identification
**1**	14.4	258,270,346	787	655(100),331(95)	Patuletin-3-glucosyl-(1-6)[apiosyl(1-2)]-glucoside
**2**	15.03	314	337	191(25),173(5),163(11),113(5)	5-*p*-Coumaroylquinic acid
**3**	16.89	256,271,345	801	651(57),345(100)	Spinacetin-3-glucosyl-(1-6)[apiosyl(1-2)]-glucoside
**4**	17.71	275,318	933	787(100),655(98),331(75)	Patuletin-3-*O*-β-d-(2-ρ-coumaroylglucopyranosyl-(1→6)- [β-d-apiofuranosyl-(1→2)]-β-d-glucopyranoside isomer
**5**	18.36	275,318	933	787(100),655(98),331(75)	Patuletin-3-*O*-β-d-(2-ρ-coumaroylglucopyranosyl-(1→6)- [β-d-apiofuranosyl-(1→2)]-β-d-glucopyranoside
**6**	19.58	253,271,226	963	787(100),655(95),331(70)	Patuletin-3-*O*-β-d-(2-feruloylglucopyranosyl)-(1→6)-[β-Dapiofuranosyl-(1→2)]-β-d-glucopyranoside
**7**	20.63	252,274,336	947	801(100),345(98)	Spinacetin-3-*O*-β-d-(2-ρ-coumaroyl glucopyranosyl-(1→6)-[β-d-apiofuranosyl-(1→2)]-β-d-glucopyranoside isomer
**8**	21.39	259,276,318	947	801(100),345(98)	Spinacetin-3-*O*-β-d-(2-ρ-coumaroyl glucopyranosyl-(1→6)-[β-d-apiofuranosyl-(1→2)]-β-d-glucopyranoside
**9**	22.2	252,275,334	977	669(60),345(100)	Spinacetin-3-(2′′-feruloylglucosyl)(1-6)[apiosyl(1-2)]-glucoside
**10**	25.14	254,271,340	521	345(100),330(5)	Spinacetin glucuronide
**11**	27.93	252,272,340	535	359(100),345(5)	Jaceidin glucuronide
**12**	31.54	252,278,340	519	343(100),328(5)	5,3′,4′-Trihydroxy-3-methoxy-6:7-methylenedioxyflavone-4′-glucuronide
**13**	33.8	249,278,340	533	357(100)	5,4′-Dihydroxy-3,3′-dimethoxy-6:7-methylenedioxiflavone-4′-glucuronide

**Table 8:** Quantification (mg/g dw) of phenolic compounds of spinach leaves in relation to genotype, biostimulant and irrigation treatment.

Peak Number*																	
Genotype	Treatment	1	2	3	4	5	6	7	8	9	10	11	12	13	TPA	TF	TPC
**Fuji**	CW–	12.8 ± 0.4d	3.51 ± 0.07k	12.9 ± 0.3a	tr	tr	7.06 ± 0.07c	tr	tr	1.95 ± 0.04d	12.0 ± 0.1g	1.70 ± 0.01e	30.4 ± 0.1f	12.1 ± 0.2d	3.51 ± 0.07k	90.9 ± 0.4c	94.4 ± 0.5e
	AMW–	7.45 ± 0.02l	2.73 ± 0.0l	4.68 ± 0.05m	tr	tr	1.64 ± 0.02k	tr	tr	tr	6.72 ± 0.02o	tr	23.51 ± 0.09kl	4.70 ± 0.02n	2.73 ± 0.06l	48.71 ± 0.04h	51.43 ± 0.02k
	MEGW–	7.8 ± 0.2j	6.58 ± 0.01g	6.48 ± 0.03j	tr	tr	1.83 ± 0.05k	tr	tr	1.22 ± 0.02f	12.94 ± 0.02f	2.224 ± 0.01c	42.0 ± 0.2c	14.9 ± 0.2b	6.58 ± 0.01g	89.4 ± 0.2c	96.0 ± 0.2e
	TAW–	8.43 ± 0.06i	2.33 ± 0.07m	7.64 ± 0.01g	tr	tr	1.27 ± 0.03l	tr	tr	tr	3.8 ± 0.1p	tr	17.3 ± 0.1m	5.86 ± 0.03l	2.33 ± 0.07m	44.2 ± 0.2i	46.5 ± 0.3l
	VW–	12.8 ± 0.1d	6.78 ± 0.04g	5.8 ± 0.1k	tr	tr	3.062 ± 0.003j	tr	tr	tr	11.3 ± 0.3h	tr	28.97 ± 0.06gh	6.5 ± 0.1k	6.78 ± 0.04g	68.5 ± 0.7f	75.3 ± 0.7g
	CW+	10.4 ± 0.2g	5.35 ± 0.03i	8.0 ± 0.2f	tr	tr	5.92 ± 0.04e	tr	tr	0.80 ± 0.03g	10.391 ± 0.008j	0.751 ± 0.005i	26.6 ± 0.2j	9.0 ± 0.3gh	5.35 ± 0.03i	71.8 ± 0.2ef	77.1 ± 0.2g
	AMW+	11.57 ± 0.04e	7.42 ± 0.02f	7.718 ± 0.002g	tr	tr	5.8 ± 0.2ef	tr	tr	0.066 ± 0.001j	10.0 ± 0.1k	0.61 ± 0.01i	23.3 ± 0.3l	10.8 ± 0.2f	7.42 ± 0.02f	69.8 ± 0.8f	77.2 ± 0.8g
	MEGW+	7.64 ± 0.02k	4.93 ± 0.01j	5.70 ± 0.07k	tr	tr	1.130 ± 0.009m	tr	tr	tr	8.8 ± 0.2l	tr	28.5 ± 0.2h	9.5 ± 0.2g	4.93 ± 0.02j	61.2 ± 0.2g	66.1 ± 0.1ij
	TAW+	15.37 ± 0.05c	9.9 ± 0.3d	10.72 ± 0.03c	tr	tr	7.98 ± 0.08b	tr	tr	1.45 ± 0.02e	13.5 ± 0.2e	2.02 ± 0.08d	29.5 ± 0.1g	11.5 ± 0.3e	9.9 ± 0.3d	92.1 ± 0.5c	102.0 ± 0.8d
	VW+	20.2 ± 0.2a	5.65 ± 0.04h	10.08 ± 0.03d	tr	0.56 ± 0.02	9.44 ± 0.08a	tr	tr	3.76 ± 0.06ab	17.7 ± 0.3c	1.38 ± 0.08f	41.3 ± 0.2c	10.5 ± 0.2f	5.65 ± 0.04h	114.8 ± 0.3b	120.5 ± 0.3c
**Viroflay**	CW–	15.9 ± 0.2b	6.794 ± 0.002g	12.42 ± 0.03b	tr	tr	5.7 ± 0.2f	tr	tr	3.90 ± 0.04a	19.16 ± 0.06b	5.20 ± 0.03a	61.4 ± 0.2a	17.50 ± 0.07a	6.79 ± 0.02g	141.2 ± 0.6a	148.0 ± 0.6a
	AMW–	7.73 ± 0.03jk	10.0 ± 0.1d	7.80 ± 0.03g	tr	tr	5.3 ± 0.2g	tr	tr	2.423 ± 0.008c	11.9 ± 0.1g	1.91 ± 0.02d	29.0 ± 0.3g	10.4 ± 0.2f	10.0 ± 0.1d	76.4 ± 0.1de	86.4 ± 0.2f
	MEGW–	9.3 ± 0.2h	3.39 ± 0.02k	8.2 ± 0.2e	tr	tr	4.367 ± 0.002i	tr	tr	tr	7.07 ± 0.05n	0.42 ± 0.01j	23.794 ± 0.005k	9.34 ± 0.06h	3.39 ± 0.02k	62.47 ± 0.02g	65.86 ± 0.05ij
	TAW–	5.3 ± 0.2n	7.28 ± 0.02f	4.6 ± 0.2m	tr	tr	1.203 ± 0.002lm	tr	tr	0.164 ± 0.001i	10.37 ± 0.05j	1.05 ± 0.01h	35.904 ± 0.005d	8.00 ± 0.06j	7.28 ± 0.02f	66.59 ± 0.02f	73.87 ± 0.05gh
	VW–	12.65 ± 0.09d	6.598 ± 0.005g	5.55 ± 0.04l	tr	tr	2.99 ± 0.01j	tr	tr	tr	11.0 ± 0.2i	tr	27.95 ± 0.03i	4.02 ± 0.02o	6.60 ± 0.05g	64.1 ± 0.2fg	70.7 ± 0.2hi
	CW+	5.33 ± 0.05n	5.03 ± 0.04j	3.6 ± 0.1n	tr	tr	tr	tr	tr	tr	7.6 ± 0.2m	tr	32.3 ± 0.1e	8.24 ± 0.03i	5.03 ± 0.04j	57.1 ± 0.2g	62.1 ± 0.1j
	AMW+	11.5 ± 0.1e	13.69 ± 0.07a	7.108 ± 0.009i	tr	tr	5.0 ± 0.1h	tr	tr	3.62 ± 0.04b	19.7 ± 0.1a	4.1 ± 0.1b	53.7 ± 0.3b	13.73 ± 0.05c	13.69 ± 0.07a	118.6 ± 0.1b	132.25 ± 0.07b
	MEGW+	10.91 ± 0.09f	11.7 ± 0.1b	7.467 ± 0.004h	tr	tr	6.2 ± 0.1d	tr	tr	0.674 ± 0.003h	14.5 ± 0.2d	1.98 ± 0.02d	32.8 ± 0.6e	8.39 ± 0.04i	11.7 ± 0.2b	82.9 ± 0.4d	94.6 ± 0.3e
	TAW+	2.91 ± 0.01o	11.0 ± 0.1c	1.617 ± 0.005o	tr	tr	tr	tr	tr	tr	10.9 ± 0.2i	1.16 ± 0.06g	27.5 ± 0.3i	5.4 ± 0.1m	11.0 ± 0.1c	49.5 ± 0.6h	60.5 ± 0.7j
	VW+	5.71 ± 0.03m	8.4 ± 0.1e	4.6 ± 0.1m	tr	tr	0.759 ± 0.002n	tr	tr	0.034 ± 0.001k	11.255 ± 0.001h	1.61 ± 0.03e	35.2 ± 0.3d	7.9 ± 0.1j	8.4 ± 0.1e	67.1 ± 0.2f	75.54 ± 0.08g

F values: F_Genotype_: 18.5; F_Water-stress_: 7.71; F_Biostimulant_: 2.79; F_Interaction_: 2.79. Degrees of freedom (DF): DF_Genotype_: 1; DF_Water-stress_: 1; DF_Biostimulant_: 4; DF_Interaction_: 4. tr-traces. W+: indicates normal irrigation regime; W–: indicates water-holding irrigation regime; C: Control; AM: Aminovert; MEG: Megafol; TA: Twin-Antistress; V: Veramin Ca. *Means in the same column followed by different Latin letters are significantly different according to Tukey’s HSD test (*p* = 0.05). TPA: Total phenolic acids; TF: Total flavonoids; TPC: Total phenolic compounds. For phenolic compounds names, refer to Table 7. Standard calibration curves used for quantification: Quercetin-3-*O*-rutinoside (*y* = 13343*x* + 76751, *R²* = 0.9998, Limit of detection (LOD) = 14.71 µg/mL and limit of quantitation (LOQ) = 44.59 µg/mL, peaks 1 and 3–13), and *p*-coumaric acid (*y* = 301950*x* + 6966.7, *R²* = 0.9999, LOD = 1.10 µg/mL and LOQ = 3.32 µg/mL, peak 2).

**Table 9:** Antioxidant activity (IC_50_, µg/mL) of spinach leaves in relation to genotype, biostimulant and irrigation treatment.

Genotype	Treatment	OxHLIA*	TBARS
**Fuji**	CW–	387 ± 11c	579 ± 2p
	AMW–	na	260 ± 13i
	MEGW–	na	267 ± 7i
	TAW–	na	94 ± 4c
	VW–	396 ± 10c	85 ± 3b
	CW+	na	563 ± 27o
	AMW+	na	202 ± 7g
	MEGW+	na	292 ± 14j
	TAW+	na	105 ± 1d
	VW+	187 ± 1b	109 ± 1d
**Viroflay**	CW–	na	522 ± 6n
	AMW–	na	141 ± 3e
	MEGW–	na	472 ± 17m
	TAW–	na	166 ± 8f
	VW–	na	346 ± 13k
	CW+	na	569 ± 10o
	AMW+	na	240 ± 12h
	MEGW+	na	283 ± 13j
	TAW+	na	147 ± 7e
	VW+	na	379 ± 16l
	Trolox	19.6 ± 0.6a	5.8 ± 0.6a

F values: F_Genotype_: 18.5; F_Water-stress_: 7.71; F_Biostimulant_: 2.79; F_Interaction_: 2.79. Degrees of freedom (DF): DF_Genotype_: 1; DF_Water-stress_: 1; DF_Biostimulant_: 4; DF_Interaction_: 4. *OxHLIA: oxidative hemolysis inhibition assay; TBARS: thiobarbituric acid reactive substances na: no activity. W+: indicates normal irrigation regime; W–: indicates water-holding irrigation regime; C: Control; AM: Aminovert; MEG: Megafol; TA: Twin-Antistress; V: Veramin Ca. Means in the same column and the same genotype and irrigation treatment followed by different Latin letters are significantly different according to Tukey’s HSD test (*p* = 0.05); The asterisk symbol (*) indicates significant differences between means of the same column and the same genotype and biostimulant treatment according to Tukey’s HSD test (*p* = 0.05).

**Table 10:** Cytotoxicity and anti-inflammatory activity (GI_50_, µg/mL).

Genotype	Treatment	NCI-H460	HeLa	HepG2	MCF-7	PLP2	RAW 264.7
**Fuji**	CW–	>400	>400	>400	>400	>400	>400
	AMW–	>400	>400	>400	>400	>400	>400
	MEGW–	>400	>400	>400	>400	>400	>400
	TAW–	>400	280 ± 22	322 ± 5	283 ± 11	>400	>400
	VW–	>400	>400	>400	>400	>400	>400
	CW+	>400	>400	>400	>400	>400	>400
	AMW+	>400	>400	>400	>400	>400	>400
	MEGW+	>400	>400	>400	>400	>400	>400
	TAW+	>400	>400	>400	>400	>400	>400
	VW+	>400	>400	>400	>400	>400	>400
**Viroflay**	CW–	>400	>400	>400	>400	>400	>400
	AMW–	>400	>400	>400	>400	>400	>400
	MEGW–	>400	339 ± 5	377 ± 17	>400	>400	>400
	TAW–	>400	>400	>400	>400	>400	>400
	VW–	>400	>400	>400	>400	>400	>400
	CW+	>400	364 ± 20	383 ± 15	>400	>400	>400
	AMW+	>400	>400	>400	>400	>400	>400
	MEGW+	>400	>400	>400	>400	>400	>400
	TAW+	>400	>400	>400	>400	>400	>400
	VW+	>400	>400	>400	>400	>400	>400
	Ellipticine	1.03 ± 0.09	1.91 ± 0.06	1.1 ± 0.2	0.91 ± 0.04	3.2 ± 0.7	-
	Dexamethaxone	-	-	-	-	-	16 ± 1

F values: F_Genotype_: 18.5; F_Water-stress_: 7.71; F_Biostimulant_: 2.79; F_Interaction_: 2.79. Degrees of freedom (DF): DF_Genotype_: 1; DF_Water-stress_: 1; DF_Biostimulant_: 4; DF_Interaction_: 4. W+: indicates normal irrigation regime; W–: indicates water-holding irrigation regime; C: Control; AM: Aminovert; MEG: Megafol; TA: Twin-Antistress; V: Veramin Ca. NCI-H460 (non-small cell lung cancer), HeLa (cervical carcinoma), HepG2 (hepatocellular carcinoma), MCF-7 (breast carcinoma), PLP2 (primary culture of porcine liver non-tumor cells), and RAW 264.7 (macrophage cell line). Means in the same column and the same genotype and irrigation treatment followed by different Latin letters are significantly different according to Tukey’s HSD test (*p* = 0.05); The asterisk symbol (*) indicates significant differences between means of the same column and the same genotype and biostimulant treatment according to Tukey’s HSD test (*p* = 0.05).

**Table 11:** Antibacterial activity (mg/mL) of spinach leaves’ extracts in relation to genotype, biostimulant and irrigation treatment.

Genotype	Treatments		S.a.	B.c.	L.m.	E.c.	S.t.	En.cl.
**Fuji**	CW–	MIC	2	1	4	1	4	4
		MBC	4	2	8	2	8	8
	AMW–	MIC	2	1	2	1	2	1
		MBC	4	2	4	2	4	2
	MEGW–	MIC	4	0.5	2	2	2	2
		MBC	8	1	4	4	4	4
	TAW–	MIC	>8	1	1	1	2	2
		MBC	>8	2	2	2	4	4
	VW–	MIC	>8	1	>8	>8	>8	>8
		MBC	>8	2	>8	>8	>8	>8
	CW+	MIC	4	0.5	1	1	1	1
		MBC	8	1	2	2	2	2
	AMW+	MIC	4	4	4	2	4	2
		MBC	8	8	8	4	8	4
	MEGW+	MIC	4	2	4	4	8	4
		MBC	8	4	8	8	>8	8
	TAW+	MIC	4	1	4	2	4	4
		MBC	8	2	8	4	8	8
	VW+	MIC	4	0.5	1	0.5	0.5	0.5
		MBC	8	1	2	1	1	1
**Viroflay**	CW–	MIC	>8	4	8	2	8	4
		MBC	>8	8	>8	4	>8	8
	AMW–	MIC	8	4	4	2	4	4
		MBC	>8	8	8	4	8	8
	MEGW–	MIC	4	1	>8	4	4	4
		MBC	8	2	>8	8	8	8
	TAW–	MIC	>8	1	2	2	2	2
		MBC	>8	2	4	4	4	4
	VW–	MIC	8	2	8	4	>8	8
		MBC	>8	4	>8	8	>8	>8
	CW+	MIC	8	0.5	1	1	0.5	0.5
		MBC	>8	1	2	2	1	1
	AMW+	MIC	4	2	4	2	>8	4
		MBC	8	4	8	4	>8	8
	MEGW+	MIC	4	0.5	1	2	1	1
		MBC	8	1	2	4	2	2
	TAW+	MIC	>8	0.5	0.5	1	1	1
		MBC	>8	1	1	2	2	2
	VW+	MIC	4	4	4	2	4	4
		MBC	8	8	8	4	8	8
	Streptomycin*	MIC	0.17	0.10	0.20	0.20	0.20	0.043
		MBC	0.25	0.20	0.30	0.30	0.30	0.25
	Ampicilin*	MIC	0.34	0.25	0.40	0.40	0.75	0.086
		MBC	0.37	0.40	0.50	0.50	1.20	0.37

F values: F_Genotype_: 18.5; F_Water-stress_: 7.71; F_Biostimulant_: 2.79; F_Interaction_: 2.79. Degrees of freedom (DF): DF_Genotype_: 1; DF_Water-stress_: 1; DF_Biostimulant_: 4; DF_Interaction_: 4. *Positive controls; ^¥^W+: indicates normal irrigation regime; W–: indicates water-holding irrigation regime; C: Control; AM: Aminovert; MEG: Megafol; TA: Twin-Antistress; V: Veramin Ca. MIC: minimum inhibitory concentration (mg/mL); MBC: minimum bactericidal concentrations (mg/mL); Bacterial strains: S.a. (*Staphylococcus aureus*; ATCC 6538), B.c.. (*Bacillus cereus*; food isolate), L.m. (*Listeria monocytogenes*; NCTC 7973), E.c. (*Escherichia coli*; ATCC 35210), S.t. (*Salmonella* Typhimurium; ATCC 13311), and En.cl. (*Enterobacter cloacae*; human isolate).

**Table 12:** Antifungal activity (mg/mL) of spinach leaves’ extracts in relation to genotype, biostimulant and irrigation treatment.

Genotype	Treatments		A.n.	A.v.	P.f.	P.v.c.	P.o.	T.v.
**Fuji**	CW–	MIC	8	8	8	>8	>8	8
		MFC	>8	>8	>8	>8	>8	>8
	AMW–	MIC	>8	>8	>8	>8	>8	>8
		MFC	>8	>8	>8	>8	>8	>8
	MEGW–	MIC	>8	>8	>8	>8	>8	8
		MFC	>8	>8	>8	>8	>8	>8
	TAW–	MIC	4	4	2	>8	>8	4
		MFC	8	8	4	>8	>8	8
	VW–	MIC	>8	>8	>8	>8	>8	8
		MFC	>8	>8	>8	>8	>8	>8
	CW+	MIC	4	4	4	4	4	4
		MFC	8	4	4	8	8	4
	AMW+	MIC	8	8	8	8	8	8
		MFC	>8	>8	>8	>8	>8	>8
	MEGW+	MIC	8	8	4	>8	>8	8
		MFC	8	8	8	>8	>8	8
	TAW+	MIC	8	8	8	8	8	8
		MFC	>8	>8	>8	>8	>8	>8
	VW+	MIC	>8	>8	>8	8	>8	8
		MFC	>8	>8	>8	>8	>8	>8
**Viroflay**	CW–	MIC	>8	>8	>8	>8	>8	>8
		MFC	>8	>8	>8	>8	>8	>8
	AMW–	MIC	2	2	2	>8	>8	2
		MFC	4	4	4	>8	>8	4
	MEGW–	MIC	>8	>8	>8	>8	>8	8
		MFC	>8	>8	>8	>8	>8	>8
	TAW–	MIC	8	8	8	>8	>8	8
		MFC	>8	>8	>8	>8	>8	>8
	VW–	MIC	>8	8	8	>8	>8	8
		MFC	>8	>8	>8	>8	>8	>8
	CW+	MIC	8	8	8	8	8	8
		MFC	8	8	8	>8	>8	8
	AMW+	MIC	8	8	8	>8	>8	8
		MFC	8	8	8	>8	>8	8
	MEGW+	MIC	4	4	4	>8	4	4
		MFC	8	8	8	>8	8	8
	TAW+	MIC	8	8	8	>8	>8	8
		MFC	>8	>8	>8	>8	>8	>8
	VW+	MIC	8	8	8	>8	>8	8
		MFC	>8	>8	>8	>8	>8	>8
	Ketoconazole*	MIC	0.20	0.20	0.20	0.20	3.8	4.75
		MFC	0.50	0.50	0.50	0.30	0.48	0.64
	Bifonazole*	MIC	0.15	0.10	0.20	0.10	3.8	5.70
		MFC	0.20	0.20	0.25	0.20	0.64	0.80

F values: F_Genotype_: 18.5; F_Water-stress_: 7.71; F_Biostimulant_: 2.79; F_Interaction_: 2.79. Degrees of freedom (DF): DF_Genotype_: 1; DF_Water-stress_: 1; DF_Biostimulant_: 4; DF_Interaction_: 4. *Positive controls; ^¥^W+: indicates normal irrigation regime; W–: indicates water-holding irrigation regime; C: Control; AM: Aminovert; MEG: Megafol; TA: Twin-Antistress; V: Veramin Ca. MIC: minimum inhibitory concentration (mg/mL); MFC: minimum fungicidal concentrations (mg/mL); Fungal strains: A.n. (*Aspergillus niger*; ATCC 6275), A.v. (*Aspergillus versicolor*; ATCC 11730), P.f. (*Penicillium funiculosum*; ATCC 36839), P.v.c. (*Penicillium aurantiogriseum (Penicillium verrucosum var. cyclopium)*; food isolate), P.o. (*Penicillium ochrochloron*; ATCC 9112), and T.v. (*Trichoderma viride*; IAM 5061).

## References

[1] Howladar S.M. (2018). Potassium humate improves physio-biochemical attributes, defense systems activities and water-use efficiencies of eggplant under partial root-zone drying. Sci. Hortic. (Amsterdam).

[2] Schonhof I., Kläring H.-P., Krumbein A., Claußen W., Schreiner M. (2007). Effect of temperature increase under low radiation conditions on phytochemicals and ascorbic acid in greenhouse grown broccoli. Agric. Ecosyst. Environ..

[3] Postel S.L. (2000). Entering an era of water scarcity: The challenges ahead. Ecol. Appl..

[4] Waśkiewicz A., Gładysz O., Beszterda M., Goliński P. (2016). Water stress and vegetable crops. Water Stress Crop Plants A Sustain. Approach.

[5] Parađiković N., Teklić T., Zeljković S., Lisjak M., Špoljarević M. (2019). Biostimulants research in some horticultural plant species—A review. Food Energy Secur..

[6] Colla G., Rouphael Y. (2015). Biostimulants in horticulture. Sci. Hortic. (Amsterdam).

[7] Colla G., Rouphael Y., Di Mattia E., El-Nakhel C., Cardarelli M. (2015). Co-inoculation of *Glomus intraradices* and *Trichoderma atroviride* acts as a biostimulant to promote growth, yield and nutrient uptake of vegetable crops. J. Sci. Food Agric..

[8] Chehade L.A., Al Chami Z., De Pascali S.A., Cavoski I., Fanizzi F.P. (2018). Biostimulants from food processing by-products: Agronomic, quality and metabolic impacts on organic tomato (*Solanum lycopersicum* L.). J. Sci. Food Agric..

[9] Rouphael Y., Franken P., Schneider C., Schwarz D., Giovannetti M., Agnolucci M., De Pascale S., Bonini P., Colla G. (2015). Arbuscular mycorrhizal fungi act as biostimulants in horticultural crops. Sci. Hortic. (Amsterdam).

[10] Petropoulos S.A., Taofiq O., Fernandes Â., Tzortzakis N., Ciric A., Sokovic M., Barros L., Ferreira I.C. (2019). Bioactive properties of greenhouse-cultivated green beans (*Phaseolus vulgaris* L.) under biostimulants and water-stress effect. J. Sci. Food Agric..

[11] Du Jardin P. (2015). Plant biostimulants: Definition, concept, main categories and regulation. Sci. Hortic. (Amsterdam).

[12] Calvo P., Nelson L., Kloepper J.W. (2014). Agricultural uses of plant biostimulants. Plant Soil.

[13] Rouphael Y., Giordano M., Cardarelli M., Cozzolino E., Mori M., Kyriacou M.C., Bonini P., Colla G. (2018). Plant-and seaweed-based extracts increase yield but differentially modulate nutritional quality of greenhouse spinach through biostimulant action. Agronomy.

[14] Rouphael Y., Kyriacou M.C., Petropoulos S.A., De Pascale S., Colla G. (2018). Improving vegetable quality in controlled environments. Sci. Hortic. (Amsterdam).

[15] Halpern M., Bar-Tal A., Ofek M., Minz D., Muller T., Yermiyahu U. (2015). The use of biostimulants for enhancing nutrient uptake. Adv. Agron..

[16] Bulgari R., Cocetta G., Trivellini A., Vernieri P., Ferrante A. (2015). Biostimulants and crop responses: A review. Biol. Agric. Hortic..

[17] Azcona I., Pascual I., Aguirreolea J., Fuentes M., García-Mina J.M., Sánchez-Díaz M. (2011). Growth and development of pepper are affected by humic substances derived from composted sludge. J. Plant Nutr. Soil Sci..

[18] Hernandez O.L., Calderín A., Huelva R., Martínez-Balmori D., Guridi F., Aguiar N.O., Olivares F.L., Canellas L.P. (2014). Humic substances from vermicompost enhance urban lettuce production. Agron. Sustain. Dev..

[19] Xu C., Leskovar D.I. (2015). Effects of *A. nodosum* seaweed extracts on spinach growth, physiology and nutrition value under drought stress. Sci. Hortic. (Amsterdam).

[20] Fiorentino N., Ventorino V., Woo S.L., Pepe O., De Rosa A., Gioia L., Romano I., Lombardi N., Napolitano M., Colla G. (2018). Trichoderma-based biostimulants modulate rhizosphere microbial populations and improve N uptake efficiency, yield, and nutritional quality of leafy vegetables. Front. Plant Sci..

[21] Manaf H.H. (2016). Beneficial effects of exogenous selenium, glycine betaine and seaweed extract on salt stressed cowpea plant. Ann. Agric. Sci..

[22] Koleška I., Hasanagić D., Todorović V., Murtić S., Klokić I., Paradiković N., Kukavica B. (2017). Biostimulant prevents yield loss and reduces oxidative damage in tomato plants grown on reduced NPK nutrition. J. Plant Interact..

[23] Ertani A., Pizzeghello D., Francioso O., Sambo P., Sanchez-Cortes S., Nardi S. (2014). *Capsicum chinensis* L. growth and nutraceutical properties are enhanced by biostimulants in a long-term period: Chemical and metabolomic approaches. Front. Plant Sci..

[24] Kunicki E., Grabowska A., Sękara A., Wojciechowska R. (2010). The effect of cultivar type, time of cultivation, and biostimulant treatment on the yield of spinach (*Spinacia oleracea* L.). Folia Hortic..

[25] Golubkina N.A., Kosheleva O.V., Krivenkov L.V., Dobrutskaya H.G., Nadezhkin S., Caruso G. (2017). Intersexual differences in plant growth, yield, mineral composition and antioxidants of spinach (*Spinacia oleracea* L.) as affected by selenium form. Sci. Hortic. (Amsterdam).

[26] Vargas-Hernandez M., Bobadilla I.M., Guevara-Gonzalez R.G., Velazquez Rosalia V.O., Garcia E.R., Gomez R.S.d., Arquieta A.L.d., Pacheco I.T. (2017). Plant Hormesis Management with Biostimulants of Biotic Origin in Agriculture. Front. Plant Sci..

[27] Fan D., Hodges D.M., Zhang J., Kirby C.W., Ji X., Locke S.J., Critchley A.T., Prithiviraj B. (2011). Commercial extract of the brown seaweed *Ascophyllum nodosum* enhances phenolic antioxidant content of spinach (*Spinacia oleracea* L.) which protects *Caenorhabditis elegans* against oxidative and thermal stress. Food Chem..

[28] Chrysargyris A., Xylia P., Anastasiou M., Pantelides I., Tzortzakis N. (2018). Effects of *Ascophyllum nodosum* seaweed extracts on lettuce growth, physiology and fresh-cut salad storage under potassium deficiency. J. Sci. Food Agric..

[29] Rouphael Y., Cardarelli M., Bonini P., Colla G. (2017). Synergistic action of a microbial-based biostimulant and a plant derived-protein hydrolysate enhances lettuce tolerance to alkalinity and salinity. Front. Plant Sci..

[30] Dudaš S., Šola I., Sladonja B., Erhatić R., Ban D., Poljuha D. (2016). The effect of biostimulant and fertilizer on “low input” lettuce production. Acta Bot. Croat..

[31] Galla N.R., Pamidighantam P.R., Karakala B., Gurusiddaiah M.R., Akula S. (2016). Nutritional, textural and sensory quality of biscuits supplemented with spinach (*Spinacia oleracea* L.). Int. J. Gastron. Food Sci..

[32] Panda V., Mistry K., Sudhamani S., Nandave M., Ojha S.K. (2017). Amelioration of Abnormalities Associated with the Metabolic Syndrome by *Spinacia oleracea* (Spinach) Consumption and Aerobic Exercise in Rats. Oxid. Med. Cell. Longev..

[33] Lomnitski L., Bergman M., Nyska A., Ben-Shaul V., Grossman S. (2003). Composition, Efficacy, and Safety of Spinach Extracts. Nutr. Cancer.

[34] Petropoulos S.A., Constantopoulou E., Karapanos I., Akoumianakis C.A., Passam H.C. (2011). Diurnal variation in the nitrate content of parsley foliage. Int. J. Plant Prod..

[35] Morales P., Ferreira I.C.F.R., Carvalho A.M., Sánchez-Mata M.C., Cámara M., Fernández-Ruiz V., Pardo-de-Santayana M., Tardío J. (2014). Mediterranean non-cultivated vegetables as dietary sources of compounds with antioxidant and biological activity. LWT - Food Sci. Technol..

[36] Barcelos C., Machado R.M.A., Alves-Pereira I., Ferreira R., Bryla D.R. (2017). Effects of substrate type on plant growth and nitrogen and nitrate concentration in spinach. Int. J. Plant Biol..

[37] Hmelak Gorenjak A., Cencič A. (2013). Nitrate in vegetables and their impact on human health. A review. Acta Aliment..

[38] Carillo P., Colla G., Fusco G.M., Dell’Aversana E., El-Nakhel C., Giordano M., Pannico A., Cozzolino E., Mori M., Reynaud H. (2019). Morphological and Physiological Responses Induced by Protein Hydrolysate-Based Biostimulant and Nitrogen Rates in Greenhouse Spinach. Agronomy.

[39] Kim M.J., Shim C.K., Kim Y.K., Ko B.G., Park J.H., Hwang S.G., Kim B.H. (2018). Effect of biostimulator *Chlorella fusca* on improving growth and qualities of chinese chives and spinach in organic farm. Plant Pathol. J..

[40] Singh S. (2016). Enhancing phytochemical levels, enzymatic and antioxidant activity of spinach leaves by chitosan treatment and an insight into the metabolic pathway using DART-MS technique. Food Chem..

[41] Fan D., Hodges D.M., Critchley A.T., Prithiviraj B. (2013). A Commercial Extract of Brown Macroalga (*Ascophyllum nodosum*) Affects Yield and the Nutritional Quality of Spinach In Vitro. Commun. Soil Sci. Plant Anal..

[42] Kulkarni M.G., Rengasamy K.R.R., Pendota S.C., Gruz J., Plačková L., Novák O., Doležal K., Van Staden J. (2019). Bioactive molecules derived from smoke and seaweed *Ecklonia maxima* showing phytohormone-like activity in *Spinacia oleracea* L.. N. Biotechnol..

[43] Petek M., Ćustić M.H., Tath N., Slunjski S., Čoga L., Pavlović I., Karažija T., Lazarević B., Cvetković S. (2012). Nitrogen and Crude Proteins in Beetroot (*Beta vulgaris* var. Conditiva) under Different Fertilization Treatments. Not. Bot. Horti Agrobot. Cluj-Napoca.

[44] Rathinasabapathi B. (2000). Metabolic engineering for stress tolerance: Installing osmoprotectant synthesis pathways. Ann. Bot..

[45] Rosa M., Prado C., Podazza G., Interdonato R., González J.A., Hilal M., Prado F.E. (2009). Soluble sugars-metabolism, sensing and abiotic stress. A complex network in the life of plants. Plant Signal. Behav..

[46] Goñi O., Quille P., Connell S.O. (2018). *Ascophyllum nodosum* extract biostimulants and their role in enhancing tolerance to drought stress in tomato plants. Plant Physiol. Biochem..

[47] Gutiérrez-Rodríguez E., Lieth H.J., Jernstedt J.A., Labavitch J.M., Suslow T.V., Cantwell M.I. (2013). Texture, composition and anatomy of spinach leaves in relation to nitrogen fertilization. J. Sci. Food Agric..

[48] Craigie J. (2011). Seaweed extract stimuli in plant science and agriculture. J. Appl. Phycol..

[49] Nuss R.F., Loewus F.A. (1978). Further Studies on Oxalic Acid Biosynthesis in Oxalate-accumulating Plants. Plant Physiol..

[50] Palmieri F., Estoppey A., House G.L., Lohberger A., Bindschedler S., Chain P.S.G., Junier P. (2019). Chapter Two - Oxalic acid, a molecule at the crossroads of bacterial-fungal interactions. Adv. Appl. Microbiol..

[51] Lee S., Choi Y., Jeong H.S., Lee J., Sung J. (2018). Effect of different cooking methods on the content of vitamins and true retention in selected vegetables. Food Sci. Biotechnol..

[52] Zeb A. (2017). A simple, sensitive HPLC-DAD method for simultaneous determination of carotenoids, chlorophylls and α-tocopherol in leafy vegetables. J. Food Meas. Charact..

[53] Huang D., Ou B., Rior R.L. (2005). The chemistry behind antioxidant capacity assays. J. Agric. Food Chem..

[54] Kellős T., Tímár I., Szilágyi V., Szalai G., Galiba G., Kocsy G. (2008). Stress hormones and abiotic stresses have different effects on antioxidants in maize lines with different sensitivity. Plant Biol..

[55] Min K., Chen K., Arora R. (2019). Short versus prolonged freezing differentially impacts freeze – thaw injury in spinach leaves: Mechanistic insights through metabolite profiling. Physiol. Plant..

[56] Elkelish A.A., Alnusaire T.S., Soliman M.H., Gowayed S., Senousy H.H., Fahad S. (2019). Calcium availability regulates antioxidant system, physio-biochemical activities and alleviates salinity stress mediated oxidative damage in soybean seedlings. J. Appl. Bot. Food Qual..

[57] Laxa M., Liebthal M., Telman W., Chibani K., Dietz K.J. (2019). The role of the plant antioxidant system in drought tolerance. Antioxidants.

[58] El-Bassiouny H.M.S., El-Monem A.A.A., Abdallah M.M.-S., Soliman K.M. (2018). Role of arbuscular mycorrhiza, α-tocopherol and nicotinamide on the nitrogen containing compounds and adaptation of sunflower plant to Water stress. Biosci. Res..

[59] Zemanová V., Pavlík M., Pavlíková D., Kyjaková P. (2015). Changes in the contents of amino acids and the profile of fatty acids in response to cadmium contamination in spinach. Plant Soil Environ..

[60] Maeda N., Yoshida H., Mizushina Y., Watson R.R., Preedy V.R.B.T.-B.F. (2010). Spinach and Health: Anticancer Effect. Bioactive Foods in Promoting Health-Fruits and Vegetables.

[61] Upchurch R.G. (2008). Fatty acid unsaturation, mobilization, and regulation in the response of plants to stress. Biotechnol. Lett..

[62] Puglisi I., Barone V., Sidella S., Coppa M., Broccanello C., Gennari M., Baglieri A. (2018). Biostimulant activity of humic-like substances from agro-industrial waste on *Chlorella vulgaris* and *Scenedesmus quadricauda*. Eur. J. Phycol..

[63] Liang Y., Kong F., Torres-Romero I., Burlacot A., Cuine S., Légeret B., Billon E., Brotman Y., Alseekh S., Fernie A.R. (2019). Branched-chain amino acid catabolism impacts triacylglycerol homeostasis in *Chlamydomonas reinhardtii*. Plant Physiol..

[64] Singh J., Jayaprakasha G.K., Patil B.S. (2018). An optimized solvent extraction and characterization of unidentified flavonoid glucuronide derivatives from spinach by UHPLC-HR-QTOF-MS. Talanta.

[65] Bergqust S.Å., Gertsson U.E., Knuthsen P., Olsson M.E. (2005). Flavonoids in baby spinach (*Spinacia oleracea* L.): Changes during plant growth and storage. J. Agric. Food Chem..

[66] Bottino A., Degl’Innocenti E., Guidi L., Graziani G., Fogliano V. (2009). Bioactive compounds during storage of fresh-cut spinach: The role of endogenous ascorbic acid in the improvement of product quality. J. Agric. Food Chem..

[67] Singh J., Jayaprakasha G.K., Patil B.S. (2017). Rapid ultra-high-performance liquid chromatography/quadrupole time-of-flight tandem mass spectrometry and selected reaction monitoring strategy for the identification and quantification of minor spinacetin derivatives in spinach. Rapid Commun. Mass Spectrom..

[68] Clifford M.N., Johnston K.L., Knight S., Kuhnert N. (2003). Hierarchical scheme for LC-MSn identification of chlorogenic acids. J. Agric. Food Chem..

[69] Clifford M.N., Zheng W., Kuhnert N. (2006). Profiling the chlorogenic acids of aster by HPLC-MSn. Phytochem. Anal..

[70] Pliakoni E.D., Nanos G.D., Gil M.I. (2010). Two-season study of the influence of regulated deficit irrigation and reflective mulch on individual and total phenolic compounds of nectarines at harvest and during storage. J. Agric. Food Chem..

[71] Parađiković N., Vinković T., Vrček I.V., Žuntar I., Bojić M., Medić-Šarić M. (2011). Effect of natural biostimulants on yield and nutritional quality: An example of sweet yellow pepper (*Capsicum annuum* L.) plants. J. Sci. Food Agric..

[72] Petrozza A., Santaniello A., Summerer S., Di Tommaso G., Di Tommaso D., Paparelli E., Piaggesi A., Perata P., Cellini F. (2014). Physiological responses to Megafol® treatments in tomato plants under drought stress: A phenomic and molecular approach. Sci. Hortic. (Amsterdam).

[73] Saa S., Del Rio A.O., Castro S., Brown P.H. (2015). Foliar application of microbial and plant based biostimulants increases growth and potassium uptake in almond (*Prunus dulcis* [Mill.] D. A. Webb). Front. Plant Sci..

[74] Cho M.J., Howard L.R., Prior R.L., Morelock T. (2008). Flavonoid content and antioxidant capacity of spinach genotypes determined by high-performance liquid chromatography/mass spectrometry. J. Sci. Food Agric..

[75] Horwitz W., Latimer G. (2005). AOAC Official methods of analysis of AOAC International. Official Methods of Analysis of AOAC International.

[76] Barros L., Pereira E., Calhelha R.C., Dueñas M., Carvalho A.M., Santos-Buelga C., Ferreira I.C.F.R. (2013). Bioactivity and chemical characterization in hydrophilic and lipophilic compounds of *Chenopodium ambrosioides* L.. J. Funct. Foods.

[77] Pereira C., Barros L., Carvalho A.M., Ferreira I.C.F.R. (2013). Use of UFLC-PDA for the analysis of organic acids in thirty-five species of food and medicinal plants. Food Anal. Methods.

[78] Bessada S.M.F., Barreira J.C.M., Barros L., Ferreira I.C.F.R., Oliveira M.B.P.P. (2016). Phenolic profile and antioxidant activity of *Coleostephus myconis* (L.) Rchb.f.: An underexploited and highly disseminated species. Ind. Crops Prod..

[79] Pereira C., Calhelha R.C., Barros L., Queiroz M.J.R.P., Ferreira I.C.F.R. (2014). Synergisms in antioxidant and anti-hepatocellular carcinoma activities of artichoke, milk thistle and borututu syrups. Ind. Crops Prod..

[80] Lockowandt L., Pinela J., Roriz C.L., Pereira C., Abreu R.M.V., Calhelha R.C., Alves M.J., Barros L., Bredol M., Ferreira I.C.F.R. (2019). Chemical features and bioactivities of cornflower (*Centaurea cyanus* L.) capitula: The blue flowers and the unexplored non-edible part. Ind. Crops Prod..

[81] Corrêa R.C.G., De Souza A.H.P., Calhelha R.C., Barros L., Glamoclija J., Sokovic M., Peralta R.M., Bracht A., Ferreira I.C.F.R. (2015). Bioactive formulations prepared from fruiting bodies and submerged culture mycelia of the Brazilian edible mushroom *Pleurotus ostreatoroseus* Singer. Food Funct..

[82] Soković M., Glamočlija J., Marin P.D., Brkić D., van Griensven L.J.L.D. (2010). Antibacterial effects of the essential oils of commonly consumed medicinal herbs using an in vitro model. Molecules.

[83] Soković M., Van Griensven L.J.L.D. (2006). Antimicrobial activity of essential oils and their components against the three major pathogens of the cultivated button mushroom, *Agaricus bisporus*. Eur. J. Plant Pathol..

